# A New FACS Approach Isolates hESC Derived Endoderm Using Transcription Factors

**DOI:** 10.1371/journal.pone.0017536

**Published:** 2011-03-09

**Authors:** Yuqiong Pan, Zhengqing Ouyang, Wing Hung Wong, Julie C. Baker

**Affiliations:** 1 Departments of Statistics, Genetics, and Developmental Biology, Stanford University, Stanford, California, United States of America; 2 Department of Biology, Stanford University, Stanford, California, United States of America; 3 Departments of Statistics, Health Research and Policy, and Biology, Stanford University, Stanford, California, United States of America; 4 Department of Genetics, Stanford University, Stanford, California, United States of America; Centro Cardiologico Monzino, Italy

## Abstract

We show that high quality microarray gene expression profiles can be obtained following FACS sorting of cells using combinations of transcription factors. We use this transcription factor FACS (tfFACS) methodology to perform a genomic analysis of hESC-derived endodermal lineages marked by combinations of SOX17, GATA4, and CXCR4, and find that triple positive cells have a much stronger definitive endoderm signature than other combinations of these markers. Additionally, SOX17^+^ GATA4^+^ cells can be obtained at a much earlier stage of differentiation, prior to expression of CXCR4^+^ cells, providing an important new tool to isolate this earlier definitive endoderm subtype. Overall, tfFACS represents an advancement in FACS technology which broadly crosses multiple disciplines, most notably in regenerative medicine to redefine cellular populations.

## Introduction

Cells in the developing embryo undergo step-wise progression toward particular fates. Understanding the details of this progression program is dependent upon marking and identifying the emerging cellular populations. In the hematopoietic system, specific cell surface markers for each developmental step have been highly successful at elucidating these stages [1], [2]. The ability to classify other developmental lineages in this rigorous manner would be a significant advance for developmental biology and for regenerative medicine, which greatly depends upon understanding and selecting pure populations of precise cellular types.

Human embryonic stem cells (hESCs) can differentiate into cells reflective of early germ layers, including mesoderm, endoderm and ectoderm [3]. While the derived cell types express batteries of markers of the *in vivo* situation, the homogeneity of these cells remains unexamined. The ability to separate subpopulations of these particular lineages is critical for developing more targeted methods for specific tissue engineering. In the case of endoderm, for example, the ability to isolate and characterize a FOXA1, FOXA2 and HNF-4α positive population, might allow the more efficient development of cultured hepatocytes [4], [5]. Despite much investigation, comprehensive cell surface markers have been difficult to identify in embryonic lineages, and thus teasing apart the stepwise progression of these lineages using Fluorescence Activated Cell Sorting (FACS) has remained difficult. Although cell surface markers have not been well characterized in these emerging cell types, transcription factors are known to specifically mark cellular lineages [4]–[8]. To date using nuclear proteins to examine cellular phenotypes has not been feasible due to limitations in technology [9].

In this report, we present a methodology that uses lineage-specific transcription factors to purify specific cellular populations by multi-channel FACS. This technology, which we term tfFACS, produces intact RNA that can be further examined to deduce the molecular nature of the cells. We applied multichannel tfFACS to examine the cellular populations that emerge upon endoderm differentiation in hESCs.

## Materials and Methods

### Cell Culture

Undifferentiated hES cells (H9) were maintained on irradiated mouse embryonic fibroblast (MEF) feeders as previously described [10]. Briefly, the H9 hES cell line was obtained from WiCell Research Institute (Madison, WI, http://www.wicell.org/). Cells were cultured in DMEM/F12 medium supplemented with 20% KnockOut serum replacement, 0.1 mM nonessential amino acids (NEAA), 2 mM L-glutamine, 0.55 mM 2-mercaptoethanol (all from Invitrogen, Carlsbad, CA, http://www.invitrogen.com) and 8 ng/ml recombinant human FGF2 (Peprotech, Rocky Hill, NJ, http://www.peprotech.com). Cultures were passaged with 200 units/ml collagenase IV (Invitrogen) at a 1∶3 split ratio every 4 days. Definitive endoderm differentiation was induced from hESCs by using activin A as previously described [11]–[13]. For differentiation, hESCs were first passaged onto dishes coated with growth factor reduced Matrigel (BD Biosciences, San Diego, CA, http://www.bdbiosciences.com) and cultured in hESC media conditioned overnight on primary MEF (CM) for 2 days. Differentiation was carried out in RPMI 1640 (Invitrogen) supplemented with Glutamax, penicillin/streptomycin and varying concentrations of defined FBS (Thermo Scientific HyClone, Logan, UT, https://www.thermoscientific.com). Before initiating differentiation, hESCs were given two brief washes in PBS (Invitrogen). In differentiation experiments, FBS concentrations were 0% for the first 24 h, 0.2% for the second 24 h, and 2.0% for subsequent days of differentiation. Recombinant human activin A (R&D Systems Inc., Minneapolis, MN, http://www.rndsystems.com) was added to the differentiation medium at 100 ng/ml, and cells were treated for 5 days, with medium changed once at day 3.

### RT-quantitative PCR

Total RNA was isolated from triplicate samples using RNeasy Plus Mini Kit (Qiagen, Duesseldorf, Germany, http://www.qiagen.com) or the Ambion Recover All nucleic acid extraction kit (optimized for fixed cells) (Applied Biosystems Ambion). The RNA concentration and purity were measured by NanoDrop (Thermo Scientific, Wilmington, DE, http://www.nanodrop.com). Only the samples with the OD A260/A280 ratio and the OD A260/A230 ratio close to value of 2.0, which indicates that the RNA is pure, were analyzed. 1 µg RNA was used for reverse transcription with random hexamers in a 20 µl reaction using SuperScript ΙΙΙ First-Strand cDNA synthesis kit (Invitrogen). PCR reactions were run using 1/20 of the cDNA per reaction, and 500 nM forward and reverse primers with iQ SYBR Green Supermix (Bio-Rad, Hercules, CA, http://www.bio-rad.com). Real-time PCR was performed using the Bio-Rad iCycler. Cycling was performed as follows: 94°C for 5 min followed by 40 cycles consisting of denaturation (95°C, 30 s), annealing (56°C, 30 s), and extension (72°C, 30 s), with a final incubation at 72°C for 10 min. Relative quantification was calculated using the comparative threshold cycle (CT) method and relative quantified values were normalized against that of housekeeping gene *cyclophilin G* (*CYCG*) [11]. PCR was performed in triplicate for each sample, and 3 independent experiments were carried out. The means and standard derivations were calculated and reported here using data from one representative experiment. Primer sequences are listed in Table S1.

### FACS Cell Fixation and CXCR4 Antibody Staining

Cells were dissociated using 0.05% trypsin-0.53 mM EDTA (Invitrogen) at 37°C for 3 min followed by neutralization in hESCs medium with serum. After washing three times in Staining Buffer [bovine serum albumin (BSA) or fetal bovine serum (FBS)] (BD Biosciences, San Diego, CA, http://www.bdbiosciences.com), 1.25×10^5^ cells were aliquoted for each antibody staining. Cells were resuspended in 200 µl of the same buffer and first Fc-blocked by treatment with 50 µl human serum supplement (Irvine Scientific, Santa Ana, CA, http://www.irvinesci.com) for 15 minutes at room temperature or on ice. Excess blocking serum should not be washed from this reaction. 1.25×10^5^ pelleted cells were fixed in 100 µl of 4% paraformaldehyde (PFA) (BD Biosciences) PBS solution at 4°C for 15 minutes. Cells were washed twice in Staining Buffer (BD Biosciences). The Fc-blocked cells were then labeled with 5 µl of anti-human CXCR4-PE antibody (with direct fluorophore conjugation, R&D Systems Inc.) and incubated for 30 min on ice. Live cells without fixation were stained directly for comparison. As a negative control for analysis, cells in a separate tube were treated in parallel with PE-labeled mouse IgG2A antibody. The results showed comparable staining for fixed and unfixed cells for the cell surface markers we have used in our experiments, including CXCR4 (Fig. S1A). This is consistent with a previous study in which when methanol was used to fix cells for CD surface marker staining [14].

### GATA4 and SOX17 Direct Fluorophore Antibody Conjugation and Two-Channel FACS Antibody Staining

For SOX17 and GATA4, direct fluorophore-conjugated antibodies were not commercially available. Goat anti-human SOX17 and GATA4 (both from R&D systems Inc.) were used, but the common serotype of these primary antibodies meant that secondary fluorescent antibodies would not distinguish between them. We therefore conjugated these primary antibodies directly to fluorophores using the Molecular Probe Zenon® antibody labeling kit as follows: Cells were fixed and blocked as described above. Cells were then permeablized using Cytofix/Cytoperm containing 1% sapanin (BD Biosciences) at room temperature or on ice for 20 minutes. During penetration, label transcription factor antibodies with different fluorescence dyes: goat anti-human GATA4, Goat anti-human SOX17 Abs were conjugated with Alexa 488 and 647 respectively by using Zenon® Goat IgG Labeling Kit from Molecular Probes, according to the manufacturer's instructions (Invitrogen). Following conjugation, each labeled antibody was titrated based on the quantitative result of two-step single staining with secondary antibody. For the formal experiment, cells were then incubated on ice for 30 min with both titrated Alexa 488 conjugated anti-human GATA4 and Alexa 647 conjugated anti-human SOX17 antibodies. Each of the Isotype- Goat IgG was also labeled and stained as a negative control.

### Three-Channel GATA4, SOX17 and CXCR4 FACS Staining

For three-way multichannel FACS with the transcription factor-GATA4, SOX17 and cell surface marker CXCR4, staining was performed as follows: After fixation and blocking, cells were labeled with mouse anti-human CXCR4-PE antibody. Cells were then washed, permeablized, and stained with Alexa 488 conjugated anti-human GATA4 and Alexa 647 conjugated anti-human SOX17 according to the staining protocol indicated as above. As negative controls, PE-conjugated normal mouse IgG (for anti-human CXCR4) and Goat IgGs (for GATA4 and Sox17) were also stained in the same manner as the corresponding antibodies. Compensation samples were prepared by staining fixed hESCs with APC-conjugated mouse anti-human SSEA4 antibody, PE-conjugated mouse anti-human SSEA4 antibody (both from R&D Systems Inc.) and Alexa 488-conjugated mouse anti-human OCT4 antibody (eBioscience, San Diego, CA, http://www.ebioscience.com) for each of the 3 channels. The cell surface marker SSEA4 and transcription factor OCT4 were stained the same as for CXCR4 and endodermal transcription factor markers-GATA4 and SOX17, respectively. To exclude nonspecific staining signals from the dead cells, cells were co-stained with LIVE/DEAD® Fixable Dead Cells Stain single-color dye (Molecular Probes, Invitrogen), in parallel with antibody staining. Compared with live cells, dead cells have 50-fold higher intensity with near-IR fluorescent reactive dye. We performed nuclear transcription factor marker staining with fixable dead cell dyes and found that dead cells produced very low signal (<10%) (Fig. S1B). Since the fluorescence signals came mainly from live cells, we concluded that contamination by dead cells was not a concern. Cells were washed twice in Staining Buffer and were analyzed using LSR 1 or LSRII (BD Bioscience) in the Stanford Shared FACS Facility. Data were analyzed using the Flowjo software (Tree Star, Inc., Ashland, Oregon, http://www.treestar.com).

### RNA Quality Optimization

Four procedures will affect the intact RNA quality: fixation, staining, sorting and RNA extraction. We harvested the stained cells at different stages to check RNA quality using Agilent 2100 Bioanalyzer (Agilent Technologies, Santa Clara, CA, http://www.home.agilent.com). Total RNA was isolated using Ambion Recover All nucleic acid extraction kit (optimized for fixed cells) (Applied Biosystems Ambion). Before checking RNA quality, the RNA concentration and purity were measured by NanoDrop described as above in the section of RT-quantitative PCR analysis. When we used the standard FACS protocol and extracted RNA from the sorted cells, the RNA from fixed and stained cells appeared to be of very poor quality, and even before sorting (Fig. S2A), consistent with previous reports in the literature [2], [15]–[21]. Since the fixation process may be a cause of the RNA degradation, we varied the fixation duration to see how it affected the RNA. The results showed that fixation was not a primary cause of RNA damage (Fig. S2B). Next we investigated the staining process. We stored cells in the regular staining buffer for different durations of time after fixation. As shown in Figure S2C, the RNA quality becomes increasingly poor as the storage period increases. This suggested that when cells were dead and penetrated, the exposed RNAs might be gradually degraded by the staining buffer, perhaps due to trace amounts of RNase. Therefore we modified the staining procedure in several ways to eliminate RNase activities: instead of using serum, cells were blocked and stained in staining buffer with BSA (100 µg/ml), RNase Inhibitor (100 U/ml), and DTT (5 mM) added. We also used RNase free water to make stain solution, and maintained very low temperature (on ice or 4°C) throughout the whole procedure. Using our new protocol, we could obtain RNA of high quality. This is demonstrated in Figure S2D where clean peaks for 18S and 28S rRNA are still evident after fixation, staining, and sorting. The fixatives which are used for intracellular marker staining, either for flow cytometry or laser capture microdissection studies, include precipitive-type fixatives such as methanol, acetone, ethanol, and cross-linking fixative-neutral-buffered formalin and paraformaldehyde (PFA). According to current studies, to both fix the intracellular proteins and keep the RNA intact, methanol, acetone, and ethanol are preferred over 4% PFA [18]–[20]. These three fixatives have been successfully used in FACS staining for intracellular phosphorylated signaling proteins [15], [16]. Conversely, for tfFACS staining, we found that 4% PFA provides higher quality results.

### FACS

**Figure 1:**
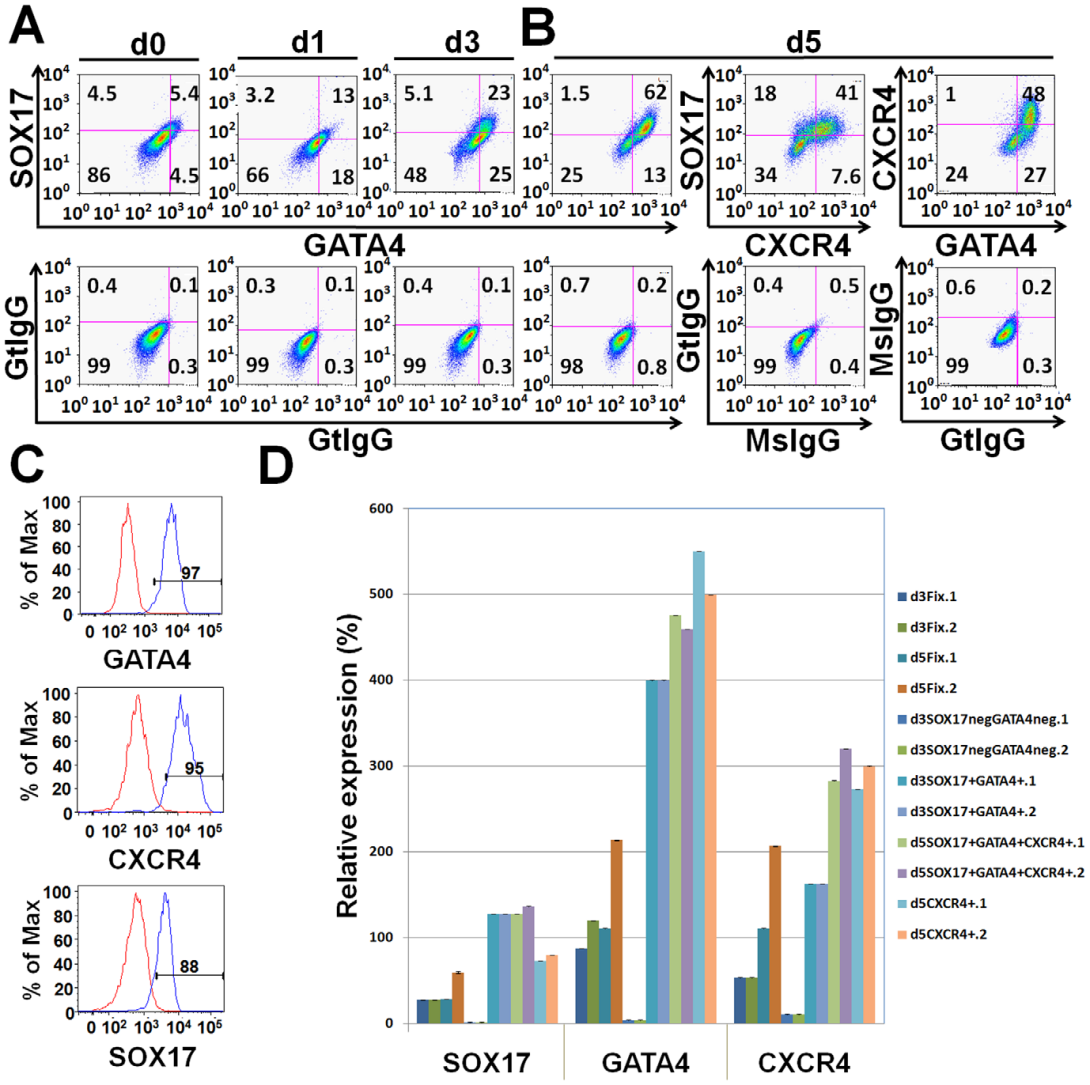
Endodermal subpopulations emerging after activin A treatment using tfFACS. (**A**) A representative experiment using two-channel FACS analysis of GATA4 and SOX17. Compared with the isotype negative control (bottom panels), three distinct cellular populations: SOX17^+^GATA4^−^, SOX17^+^GATA4^+^, and SOX17^−^GATA4^+^ are emerging gradually upon differentiation: at day 1, 13% are SOX17^+^GATA4^+^, increasing to 23% by day 3. Another significant population consists of 18% SOX17^−^GATA4^+^ at day 1 and 25% at day 3. (**B**) After 5 days of differentiation, using three-way multichannel FACS analysis for SOX17, GATA4, and CXCR4, we found that the SOX17^+^GATA4^+^ population dominates the culture (62%) and CXCR4 is expressed in 49% of the cells, most of which are SOX17^+^GATA4^+^CXCR4^+^ (41%). There are also approximately 27% GATA4^+^CXCR4^−^ cells, which comprises the population of SOX17^+^GATA4^+^CXCR4^−^ cells (21%). (**C**) Post sorting, FACS analysis demonstrated that 97% of day 5 SOX17^+^GATA4^+^CXCR4^+^ cells were positive for GATA4, 88% were SOX17 positive, and 95% were CXCR4 positive. This was consistent over 5 separate experiments. (**D**) Expression analysis using RT-qPCR demonstrates that day 5 SOX17^+^GATA4^+^CXCR4^+^ and day3 SOX17^+^GATA4^+^ cells have higher level of expression of SOX17, GATA4 and CXCR4 than unsorted fixed cells or day 3 SOX17^−^GATA4^−^ (d3SOX17negGATA4neg) cells.

When cells are prepared for sorting, two way or three way tfFACS staining was performed following the protocols above using the improved RNA conditions. d5CXCR4^+^ sorting was performed on live cells. Isotype controls were used to gate the cells (Fig. 1A, B and Fig. S1C). Sorting was performed using Aria (BD Bioscience) in the Stanford Shared FACS Facility. Sorting was done at 4°C. Cells were collected into tubes with RNase free PBS. We performed the purity checking of the sorted cells immediately after FACS separation (Fig. 1C). All the cells either from sorted populations or from the presorted mixtures were centrifuged at 14,000 rpm, 2 min, 4°C to get cell pellets. Total RNA was isolated using Ambion Recover All nucleic acid extraction kit (optimized for fixed cells) (Applied Biosystems Ambion, Austin, TX, http://www.ambion.com).

### Microarray Analysis

Samples collected after 5 days of differentiation included SOX17^+^GATA4^+^ CXCR4^+^ cells, unfixed CXCR4^+^ cells, and unsorted fixed cells. Samples collected after 3 days of differentiation included SOX17^+^GATA4^+^ cells, SOX17^−^GATA^−^ cells, and unsorted fixed cells. As controls we also collected fixed, stained hESCs using the same SOX17 GATA4 CXCR4 three-channel protocol, but without sorting. Unfixed hESCs Exon array data using the same protocol were also analyzed together [10]. All of these samples contained biological replicates, triplicates or quadruplicates. Total RNA was extracted using the Ambion Recover All nucleic acid extraction kit (optimized for fixed cells) (Applied Biosystems Ambion). Probes for the Affymetrix human Exon Array ST 1.0 were prepared and hybridized to the array using the GeneChip Whole Transcript Sense Target Labeling Assay (Affymetrix) according to the manufacturer's suggestions [10]. Briefly, for each sample, 1.5 g of total RNA was subjected to ribosomal RNA reduction. Following rRNA reduction, double-stranded cDNA was synthesized with random hexamers tagged with a T7 promoter sequence. The double-stranded cDNA was used as a template for amplification with T7 RNA polymerase to create antisense cRNA. Next, random hexamers were used to reverse transcribe the cRNA to produce single-stranded sense strand DNA. The DNA was fragmented and biotin labeled. The probes of all samples (H9 passages 40–55) were hybridized to the Affymetrix Exon Array ST 1.0 microarrays and scanned.

### Expression Data Processing

We computed gene expression indices for all the samples analyzed using the GeneBASE software [22]. Specifically, correction for background noise was performed for every core probe using the adapted MAT model of background probes in Affymetrix Exon Arrays. The background-corrected intensities were normalized across arrays by core-probe-scaling so that the median intensity of core probes in each sample was equal to 100. The normalized probe intensities were then summarized to gene level expression indices based on the dChip model [23]. The gene expression indices across arrays were quantile-normalized to generate the final gene expression profiles. The clustering heatmap was generated by dChip using the default setting, i.e, the “1-correlation” distance metric and the centroid linkage method. The raw data files have been deposited in the Gene Expression Omnibus (GEO) database with accession number GSE24135.

## Results

### tfFACS Allows Isolation of Cells Expressing Combinations of SOX17 and GATA4

**Figure 2:**
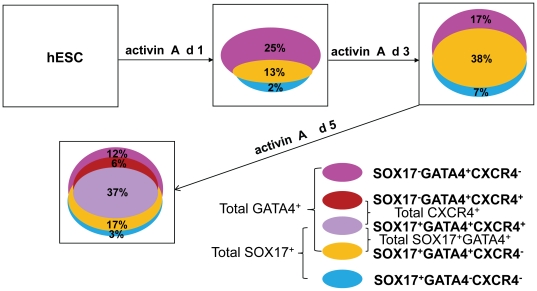
Venn diagram cartoon summarizing data obtained from 4 independent experiments which were averaged. The color key is represented on the lower right.

hESCs can differentiate into endodermal cells by dosing with high levels of the NODAL signaling pathway, but it remains unknown whether this differentiation results in several endodermal cell sub-types or a single homogeneous population. We sought to isolate and characterize these resulting endodermal cells. To this end, we differentiated hESCs into endoderm using activin A in low serum conditions [11]–[13]. Over the five days of differentiation, consistent with the observations of others, we found that markers of mesendoderm, including *BRACHYURY* are transiently expressed at 24 hours, and markers of endoderm, including *SOX17* and *GATA4*, become highly expressed at 3 and 5 days post-differentiation (Fig. S3) [6]–[8], [24], [25]. The expression of these transcription factors, allowed us to develop multichannel tfFACS using antibodies against SOX17 and GATA4. To this end, hESCs derived endodermal cells 5 days post differentiation were fixed, processed and examined for RNA quality. While multiple conditions were investigated, most of these led to massive RNA degradation, consistent with previous reports (Fig. S2A) [18]–[21]. We found that the single most influential factor was not extent of fixation, but the amount of time the sample is stored following fixation (See Materials and Methods for details and Fig. S2B, C). Briefly, cells were fixed with 4% paraformaldehyde at 4°C for 15 min, and stained using both anti-human GATA4 and anti-human SOX17 antibodies conjugated with the fluorescence dyes-Alexa 488 and 647, respectively. As negative controls for analysis, normal goat IgG antibody was also conjugated with Alexa 488 and 647. Stained cells were then analyzed using two-channel FACS. We found three distinct cellular populations in hESC derived endoderm after 5 days of differentiation: SOX17^+^GATA4^−^, SOX17^+^GATA4^+^ and SOX17^−^GATA4^+^ (Fig. 1A, B and 2). This observation demonstrates that treatment with activin A causes hESCs to differentiate into molecularly distinct subpopulations of endoderm.

### tfFACS Can Be Used with Combinations of Antibodies Against Transcription Factors and Cell Surface Proteins

To further investigate the extent of heterogeneity in the endodermal culture, we followed the subpopulations through the differentiation time course by adding an additional marker, CXCR4 [26]. We chose CXCR4 as the third marker because it is one of the few cell surface markers used to isolate definitive endoderm from mouse and human ESCs [11], [27]. We examined hESC-derived endoderm after a 1, 3 or 5 days of differentiation using three-way multichannel FACS analysis for SOX17, GATA4 and CXCR4, or two-way multichannel FACS analysis for SOX17 and GATA4. FACS analysis immediately following sorting to check the purity showed that 95% of the day 5 SOX17^+^GATA4^+^CXCR4^+^ cells were positive for GATA4, 90% were positive for SOX17 and more than 95% were positive for CXCR4, suggesting efficacy of the sorting protocol (Fig. 1C). To further validate the sorted populations, we performed marker analysis using RT-qPCR for *GATA4*, *SOX17* and *CXCR4*. Compared to day 3 and day 5 fixed cells, which are highly heterogeneous mixtures of differentiating cells, the day 5 SOX17^+^GATA4^+^CXCR4^+^, and the day 3 SOX17^+^GATA4^+^ express these transcripts at a much higher level, consistent with an increase of purity (Fig. 1D). Overall, we found that, during the first 24 hours of differentiation, GATA4^+^ cells increase substantially, and approximately 13% of these are also SOX17^+^. However, by day 3, the double SOX17^+^GATA4^+^ population becomes the predominant marked population (Fig. 1A, 2) and dominates the culture by day 5 (>50%) (Fig. 1B, 2). SOX17^+^GATA4^−^ cells are rare throughout the timecourse, strongly suggesting that if a cell is SOX17^+^, GATA4^+^ will also be present. By day 5, CXCR4 is expressed in approximately 43% of the cells. Interestingly, this population does not entirely overlap with that of SOX17^+^GATA4^+^ (Fig. 1B, 2), suggesting that the diversity of cells after treatment with activin A is greater than previously thought. This indicates that experiments using CXCR4 to isolate definitive endoderm may have missed the SOX17^+^GATA4^+^CXCR4^−^ cells, which comprise about 17% of the total population.

### tfFACS Does Not Substantially Alter Gene Expression

In order to further elucidate the molecular nature of these endodermal populations, we first needed to show that tfFACS does not alter gene expression due to the fixation protocol. Initially, we examined both hESCs and derived endoderm, either fixed or unfixed for the expression of lineage specific markers. No difference in expression levels of *OCT4* (hESCs) or *SOX17*, *GATA4*, *or CXCR4* (derived endoderm) were observed between fixed and unfixed cells (Fig. S2E). We next measured global gene expression using microarray technology on cells sorted using tfFACS. Samples collected after 5 days of differentiation included SOX17^+^GATA4^+^ CXCR4^+^ cells, unfixed CXCR4^+^ cells, and unsorted fixed cells. Samples collected after 3 days of differentiation included SOX17^+^GATA4^+^ cells, SOX17^−^GATA^−^ cells, and unsorted fixed cells. As controls, we analyzed both unfixed hESCs and fixed hESCs [10]. All samples contained biological duplicates, triplicates or quadriplicates (Fig. S4). We then performed hierarchical clustering to demonstrate whether cellular fixation alone could change gene expression. We based this analysis on 1647 transcript clusters with coefficient of variation >0.5 across the samples and expression values > = 500 in at least 2 out of the 21 samples. We found that the degree of distortion due to fixation is small particularly when compared between samples and stages. Two illustrations of this are that, first, fixed and unfixed cells cluster together based upon differentiation stage, not based upon degree of fixation, second, even though hESC and d5CXCR4^+^ are unfixed, unstained samples, they do not cluster together. Instead, each is clustered with the fixed samples that are biologically similar: hESCs with fixed hESC cells, and d5 CXCR4^+^ cells with fixed day 5 samples (Fig. S4).

### SOX17^+^GATA4^+^CXCR4^+^ is enriched for Definitive Endodermal Transcripts

**Figure 3:**
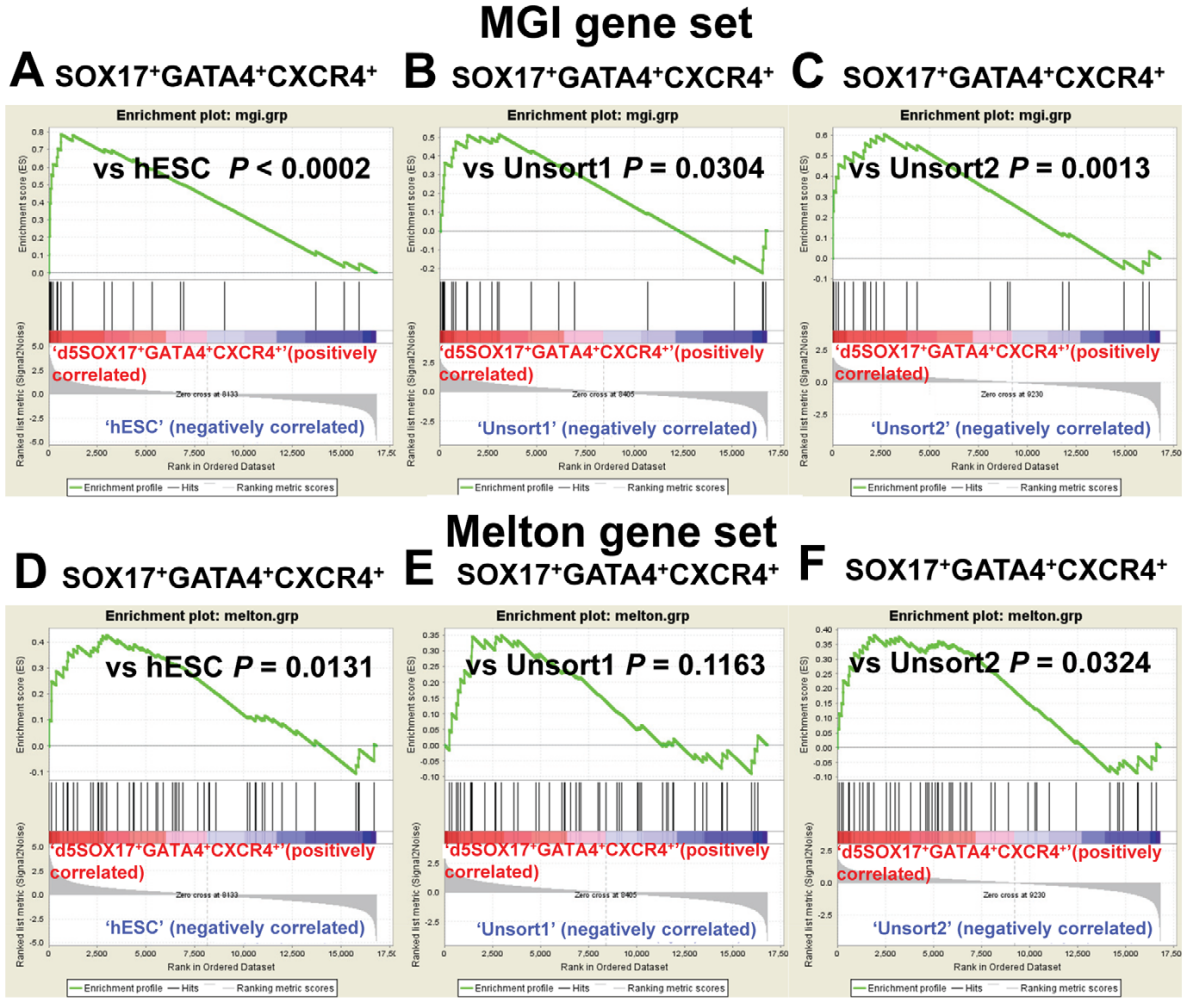
GSEA analysis of the definitive endoderm (DE) gene sets for the day 5 SOX17^+^GATA4^+^CXCR4^+^ group. As shown in (**A–C**), the MGI gene set is highly enriched in the d5 SOX17^+^GATA4^+^CXCR4^+^ cells in multiple comparisons. d5SOX17^+^GATA4^+^CXCR4^+^ vs. hESC (unfixed hESCs+fixed hESCs): P<0.0002 (**A**); d5SOX17^+^GATA4^+^CXCR4^+^ vs. Unsort1 (d3Fix+d3 SOX17^−^GATA^−^+d5Fix.1+d5Fix.3): P = 0.0304 (**B**); and d5 SOX17^+^GATA4^+^CXCR4^+^ vs Unsort2 (d5Fix.2+d5Fix.4): P = 0.0013 (**C**). We repeated the GSEA analysis on the Melton gene set. This gene set is enriched in d5SOX17^+^GATA4^+^CXCR4^+^ cells in all comparisons (**D–F**).

Our tfFACS analysis, showing discrete subpopulations with defined markers, strongly suggests that hESC derived endoderm comprises cells already specified toward particular endodermal fates. Since tissue engineering of endodermal organ systems is still in its infancy, our aim was to determine the endodermal character of each isolated population and then examine whether these subpopulations represented more specialized endodermal tissue types. To this end, we first sought to determine whether the subpopulations could be classified as definitive endoderm. Because a reliable set of human definitive endodermal marker genes has not been established, we compiled ”gold-standard” definitive endoderm gene sets: one from the Mouse Genome Informatics (MGI Set) database based on RNA in situ hybridization or immunohistochemistry evidence in E7.0–8.0 mouse (http://www.informatics.jax.org; 22 genes) and another from Sherwood et al., (Melton Set) based upon microarray profiling of E8.25 mouse definitive endoderm (51 genes, see Table 1) [28]. To determine whether these ‘gold-standard genes’ are present in the subpopulations at a level significantly higher than reference, we employed the GSEA algorithm [29]. We first compared the SOX17^+^GATA4^+^CXCR4^+^ isolated from day 5 with all the other samples, with the exception of SOX17^+^GATA4^+^ cells from day 3 and CXCR4^+^ cells from day 5, which would have extensive overlap. *As* shown in Figure 3A–C, the MGI gene set is highly enriched in the SOX17^+^GATA4^+^CXCR4^+^ day 5 sorted cells in multiple comparisons (d5 SOX17^+^GATA4^+^CXCR4^+^ vs hESC: P<0.0002; d5 SOX17^+^GATA4^+^CXCR4^+^ vs Unsort1: P = 0.0304; and d5 SOX17^+^GATA4^+^CXCR4^+^ vs Unsort2: P = 0.0013. The Unsort1 represents d3Fix+d3 SOX17^−^GATA^−^+d5Fix.1+d5Fix.3, and Unsort2 represents d5Fix.2+d5Fix.4). We repeated the GSEA analysis on the Melton gene set. Again, this gene set is enriched in d5 SOX17^+^GATA4^+^CXCR4^+^ in all comparisons (Fig. 3D–F).

**Table 1:** Definitive endoderm (DE) gene sets used in the analyses.

Gene set	Number of genes	Gene Name
MGI	22	CER1, GALNAC4S-6ST, CLDN4, CPM, DKK1, EDA, EFNA1, EMB, FOXA2, HHEX, HNF1B, ITGA3, JARID1B, LAMA1, PRDM1, SDC1, SHH, TMEM46, SOX17, TES, TMPRSS2, TRH
Melton	51	SOX17, FOXC1, GATA3, PAX6, FOXA1, EVX1, IRX3, ZHX2, PAX1, DLX5, HOXB9, RIPK4, SP6, ISL1, IRX5, SOX21, DMRTA1, PAX8, SIX3, HOXD9, PAX9, MEOX1, HOXC4, HOXA9, FOXC2, HOXB2, T, HOXB3, PAX3, PKNOX2, DLX3, DLX2, SIX1, TPBG, HOXC8, HOXD8, RFX3, CDX4, HOXA3, SOX9, HOXB1, ARNT2, HOXD1, HOXA1, FOXG1, GLI3, SOX11, IRX2, HEY2, SSBP2, PBX1

We then asked whether the SOX17^+^GATA4^+^CXCR4^+^ day 5 cells and day 3 SOX17^+^GATA4^+^ were more enriched for ‘gold-standard’ endodermal genes than the CXCR4^+^ day 5 population, which has generally been used to isolate hESC-derived endoderm [11]. To this end, we performed GSEA analysis to compare SOX17^+^GATA4^+^CXCR4^+^, SOX17^+^GATA4^+^, and CXCR4^+^ to the control group, which were all other samples combined. While both the MGI set and Melton set are enriched in both SOX17^+^GATA4^+^CXCR4^+^ and CXCR4^+^, we observed higher enrichment levels in the SOX17^+^GATA4^+^CXCR4^+^ in both comparisons (MGI: P<0.0002 and P = 0.0038, respectively; Melton: P = 0.0057 and P = 0.0105, respectively) (Fig. S5). Furthermore, day 3 SOX17^+^GATA4^+^ cells have a similar enrichment in the MGI set as day 5 SOX17^+^GATA4^+^CXCR4^+^ (P<0.0002). Importantly, the above analyses suggest that triple selection using SOX17^+^GATA4^+^CXCR4^+^ and double selection of SOX17^+^GATA4^+^ produce a more homogenous population of DE cells than selection using CXCR4 alone, which may deduce that protocols using a single FACS channel with CXCR4 are mixed with other lineages, or missing a valuable population of definitive endodermal cells. Additionally, we showed that day 3 SOX17^+^GATA4^+^ cells can be obtained at a much earlier stage of differentiation, prior to expression of CXCR4^+^ cells, providing an important new tool to isolate this earlier definitive endoderm subtype.

### Isolated Populations Are Associated With Biological Processes

To determine whether these endodermal subpopulations were indeed already fated toward specific endodermal fates, we sought to identify functional signatures using GO [30]. To this end, we selected high value representative genes from each sorted cellular population (upregulated with fold change >3 and expression values difference >100 compared to control samples). With these criteria, we selected 331 genes from the SOX17^+^GATA4^+^CXCR4^+^ day 5 cells, 442 from the CXCR4^+^ day 5 cells and 197 from the SOX17^+^GATA4^+^ day 3 cells. DAVID analysis on these groups yielded similar annotations consisting of significant biological process terms including terms “pattern specification process”, and “gastrulation” (Table S2, S3, S4, S5, S6, S7). As these annotations are shared between the sorted populations, we asked whether they arose from overlap between the sets. Comparing the gene lists from the SOX17^+^GATA4^+^CXCR4^+^ and CXCR4^+^ day 5 cells, we found that 197 genes are shared, demonstrating that overlap between these populations is extensive. DAVID analysis of these shared 197 genes is again significantly enriched in biological processes such as pattern specification process and cell morphogenesis (Table S8, S9). Unexpectedly, the genes unique to d5 CXCR4^+^ (241 genes) annotate as being significant for blood vessel morphogenesis and nervous system development (Table S10, S11) whereas those unique to SOX17^+^GATA4^+^CXCR4^+^ (129 genes) annotate as being significant only for cell adhesion (Table S12, S13). This data suggests that while there overlap as may be expected due to the use of CXCR4 in each sort, there are also distinct differences between these populations.

## Discussion

While the transcriptome of whole organisms, organ systems and culture regimes, have been described, the extent of the molecular similarities of cells within these complex groups is far from understood. This distinction is critical, as differentiating cellular populations must contain rapidly diversifying cellular types. Distinguishing between these subtle varieties of cell types is central toward a more complex biological investigation of single cell differences within these larger systems. For example, based upon transcriptional profiling it is clear that Human embryonic stem cells can differentiate into definitive endodermal cells, but based upon what we understand from the embryo these cells are unlikely to be a purely homogeneous population [3], [11], [24], [25]. For regenerative medicine and for a developmental understanding, it is important that these subtypes be isolated and characterized further.

Definitive Endoderm cells show a remarkable versatility in serving as the precursor to a multitude of cell types that constitute the visceral organs [3], [6], [28]. Using the technology described in this report, transcription factors can now be used to define populations emerging from human Embryonic Stem Cells, filling an urgent need to classify intermediate steps of differentiation. While tfFACs represents a new methodology to isolate and characterize similar cellular types from a complex mixture, it does not allow continued growth of sorted cells and thus their lineage specific commitments cannot be readily assessed. Regardless, this new method does provide a means to examine new subtypes genomically, opening up the potential for discovery of new cell surface markers and for elucidating previously uncharacterized cellular populations. As the approach has the potential to scale up to 11 channels, it could prove an unparalleled means to define cellular populations [1]. Using this approach, we find that definitive endoderm derived from hESCs is not a homogeneous population of cells, but rather diverse. We find cells within the differentiating cellular population express SOX17, GATA4 and CXCR4 together or in all possible combinations, suggesting that differing lineage potentials exist within the culture of endoderm.

Overall, this represents an advance in FACS technology that can be used to evaluate specific subpopulations and avoids the *a priori* need for lineage-specific cell surface markers, an unfulfilled need that has limited our understanding of lineage differentiation from embryonic stem cells as well as in a multitude of other disciplines, including cancer biology. The use of tfFACS to characterize lineage commitment in a systematic step wise fashion will provide inroads into understanding the molecular nature of *in vitro* derived cellular populations.

## Supporting Information

Figure S1 **Methods to identify whether fixation will affect cell surface marker staining, whether to exclude nonspecific dead cell signals from fixed cells, and how the cell sorting was performed.** (**A**) To test if fixation distorts cell surface marker staining, live and 4% paraformaldehyde (PFA) fixed (4°C, 15 min) day 5 differentiating cells were stained with PE-conjugated anti-human CXCR4 antibody, based on its negative isotype control mouse IgG (blue histogram), comparable CXCR4 staining result was detected (red histogram). Day 5 CXCR4^+^ sorting was performed on live cells. (**B**) To exclude nonspecific fluorescence from dead cell, we performed nuclear TF SOX17 staining with fixable dead cell dyes. By comparison to the isotype negative control GtIgG (bottom panel), we found that dead cells produced very low signal when sorted for Sox17 (5.47%, upper right quadrant), while the vast majority of Sox17 positive signals are from live cells (53%, lower right quadrant). (**C**) According to isotype controls, day 5 CXCR4^+^ (orange), CXCR4^−^ (purple), and SOX17^+^GATA4^+^ (box in bottom panel) cells were gated. Based on CXCR4^+^ and CXCR4^−^ subsets, day 5 SOX17^+^GATA4^+^CXCR4^+^ (blue) and SOX17^+^GATA4^+^CXCR4^−^ (green) populations were selected respectively.
(DOC)

Figure S2 **The tfFACS method used produce intact RNA following fixation, nuclear staining and FACS sorting.** (**A**) When we used the standard FACS protocol, extracted and amplified RNA from the sorted cells, the RNA from fixed and stained cells appeared to be of very poor quality measured by Agilent bioanalyzer, compared with unfixed and unstained cells. (**B**) When we varied the fixation duration from 5 min to 10 min or 15 min, we found that fixation was not a primary cause of RNA damage. Relatively intact RNA can be obtained from cells fixed by 4% paraformaldehyde at 4°C for 15 min at a level similar to that of cells fixed for 5 min and 10 min. (**C**) We stored the cells in the regular staining buffer for different amount of time after fixation. The RNA quality becomes increasingly poor as the storing period increases from 24 hours to 4 months at 4°C. (**D**) After modifying the staining procedure in several ways, we could obtain intact RNA which has clean peaks for 18S and 28S rRNA after fixation, staining and sorting. (**E**) Fixed and unfixed samples were examined by RT-qPCR analysis to determine expression levels of *OCT4* (hESCs) and *SOX17*, *GATA4*, *and CXCR4* (day 5 endoderm).
(DOC)

Figure S3 **Molecular examination of endodermal differentiation from hESCs over the course of 5 days.** RT-qPCR analysis showed that markers of endoderm, including *SOX17*, *GATA4*, and *CXCR4* become highly expressed at day 3 and day 5 post-differentiation, while *BRACHYURY (BRACH)*, a mesendodermal marker, is expressed transiently at day 1. hESCs have very low expression of endodermal genes. The cells are not expressed *SOX1*, a neuroectoderm marker throughout the timecourse. X-axis indicates days of endodermal differentiation by activin A; numbers on the Y-axis indicate relative gene expression level, normalized to that of *cyclophilin*G (*CYCG*). qPCR was performed using triplicates for each sample, and 3 independent experiments were carried out. Error bars indicate standard derivations which were calculated and reported here using data from one representative experiment.
(DOC)

Figure S4 **Hierarchical cluster shows that fixatives do not substantially change expression of cell types.** We performed hierarchical clustering and found that fixed and unfixed cells cluster together based upon cellular character, and not due to methodology. For example, hESC and d5CXCR4^+^, which have not been processed, do not cluster together, but clustered with the fixed samples that are biologically similar: hESCs with fixed hESC cells, and d5 CXCR4^+^ cells with fixed day 5 samples.
(DOC)

Figure S5 **Comparing the definitive endoderm (DE) gene set expression in SOX17^+^GATA4^+^CXCR4^+^ day 5 cells, SOX17^+^GATA4^+^ day 3 cells and day 5 CXCR4^+^ cells using GSEA analysis.** We performed GSEA analysis to compare these three populations to the control group, which are all the combined rest samples. While both the MGI DE set and Melton DE set were enriched in both d5 SOX17^+^GATA4^+^CXCR4^+^ and d5 CXCR4^+^ cells, we observed higher enrichment levels in the d5SOX17^+^GATA4^+^CXCR4^+^ population in both comparisons. MGI: P<0.0002 (**A**) and P = 0.0038 (**C**); Melton: P = 0.0057 (**D**) and P = 0.0105 (**F**). Interestingly, d3SOX17^+^GATA4^+^ cells have similar DE gene sets enrichment to d5 SOX17^+^GATA4^+^CXCR4^+^ cells (**B, E**).
(DOC)

Table S1 **Primers used for RT-qPCR analysis.**
(DOC)

Table S2 **Enrichment of top gene categories in the d5 SOX17^+^GATA4^+^CXCR4^+^ cells.**
(DOC)

Table S3 **Genes in each enriched category from d5 SOX17^+^GATA4^+^CXCR4^+^ cells.**
(DOC)

Table S4 **Enrichment of top gene categories in the d5 CXCR4^+^ cells.**
(DOC)

Table S5 **Genes in each enriched category from d5 CXCR4^+^ cells.**
(DOC)

Table S6 **Enrichment of top gene categories in the d3 SOX17^+^GATA4^+^ cells.**
(DOC)

Table S7 **Genes in each enriched category from the d3 SOX17^+^GATA4^+^ cells.**
(DOC)

Table S8 **Enrichment of top gene categories in the overlapping 197 genes from the d5 SOX17^+^GATA4^+^CXCR4^+^ and d5 CXCR4^+^ cells.**
(DOC)

Table S9 **Genes in each enriched category with overlapping 197 genes from the d5 SOX17^+^GATA4^+^CXCR4^+^ and d5 CXCR4^+^ cells.**
(DOC)

Table S10 **Enrichment of top gene categories in the unique 241 genes from the d5 CXCR4^+^ cells.**
(DOC)

Table S11 **Genes in each enriched category with the unique 241 genes from d5 CXCR4^+^ cells.**
(DOC)

Table S12 **Enrichment of top gene categories in the unique 129 genes from the d5 SOX17^+^GATA4^+^CXCR4^+^ cells.**
(DOC)

Table S13 **Genes in each enriched category with the unique 129 genes from the d5 SOX17^+^GATA4^+^CXCR4^+^ cells.**
(DOC)
