# Supplementary figures and images for: A New FACS Approach Isolates hESC Derived Endoderm Using Transcription Factors
Source: PLoS One. 2011 Mar 9;6(3):e17536. doi: 10.1371/journal.pone.0017536 (PMC3052315; doi:10.1371/journal.pone.0017536)

**Figure S1**


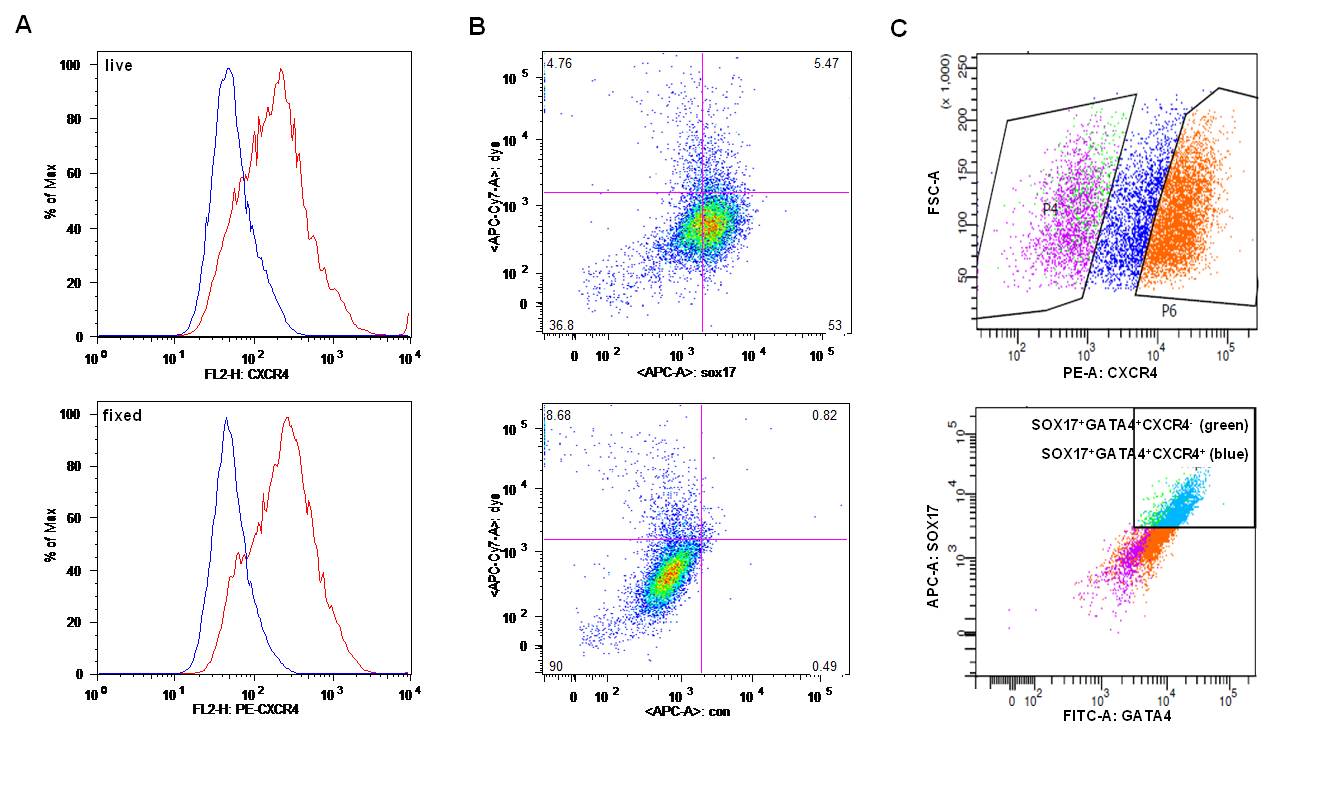

Supplement: Figure S1 — Methods to identify whether fixation will affect cell surface marker staining, whether to exclude nonspecific dead cell signals from fixed cells, and how the cell sorting was performed. (A) To test if fixation distorts cell surface marker staining, live and 4% paraformaldehyde (PFA) fixed (4°C, 15 min) day 5 differentiating cells were stained with PE-conjugated anti-human CXCR4 antibody, based on its negative isotype control mouse IgG (blue histogram), comparable CXCR4 staining result was detected (red histogram). Day 5 CXCR4+ sorting was performed on live cells. (B) To exclude nonspecific fluorescence from dead cell, we performed nuclear TF SOX17 staining with fixable dead cell dyes. By comparison to the isotype negative control GtIgG (bottom panel), we found that dead cells produced very low signal when sorted for Sox17 (5.47%, upper right quadrant), while the vast majority of Sox17 positive signals are from live cells (53%, lower right quadrant). (C) According to isotype controls, day 5 CXCR4+ (orange), CXCR4− (purple), and SOX17+GATA4+ (box in bottom panel) cells were gated. Based on CXCR4+ and CXCR4− subsets, day 5 SOX17+GATA4+CXCR4+ (blue) and SOX17+GATA4+CXCR4− (green) populations were selected respectively. (DOC) [file pone.0017536.s001.doc]

**Figure S2**


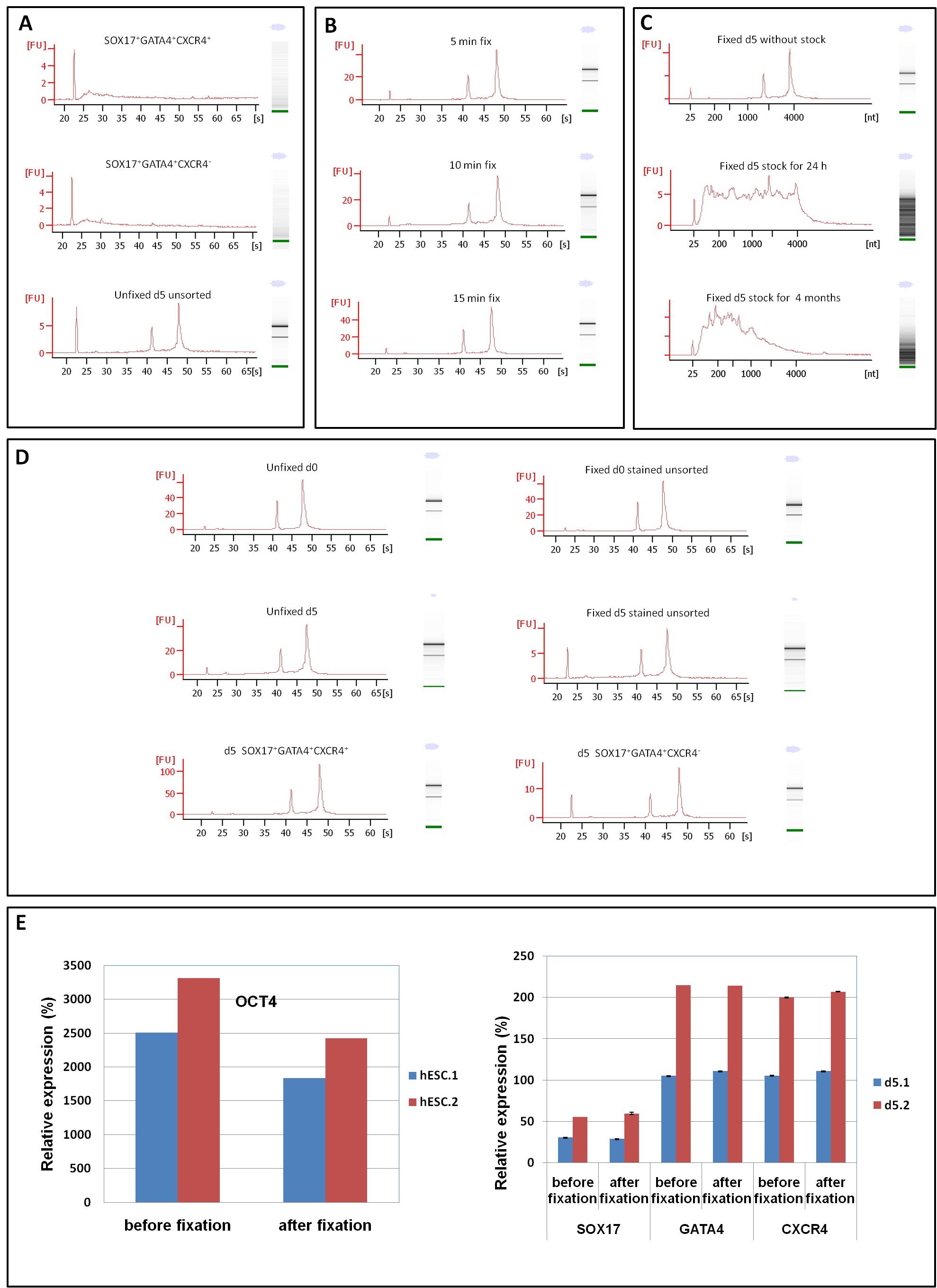

Supplement: Figure S2 — The tfFACS method used produce intact RNA following fixation, nuclear staining and FACS sorting. (A) When we used the standard FACS protocol, extracted and amplified RNA from the sorted cells, the RNA from fixed and stained cells appeared to be of very poor quality measured by Agilent bioanalyzer, compared with unfixed and unstained cells. (B) When we varied the fixation duration from 5 min to 10 min or 15 min, we found that fixation was not a primary cause of RNA damage. Relatively intact RNA can be obtained from cells fixed by 4% paraformaldehyde at 4°C for 15 min at a level similar to that of cells fixed for 5 min and 10 min. (C) We stored the cells in the regular staining buffer for different amount of time after fixation. The RNA quality becomes increasingly poor as the storing period increases from 24 hours to 4 months at 4°C. (D) After modifying the staining procedure in several ways, we could obtain intact RNA which has clean peaks for 18S and 28S rRNA after fixation, staining and sorting. (E) Fixed and unfixed samples were examined by RT-qPCR analysis to determine expression levels of OCT4 (hESCs) and SOX17, GATA4, and CXCR4 (day 5 endoderm). (DOC) [file pone.0017536.s002.doc]

**Figure S3**


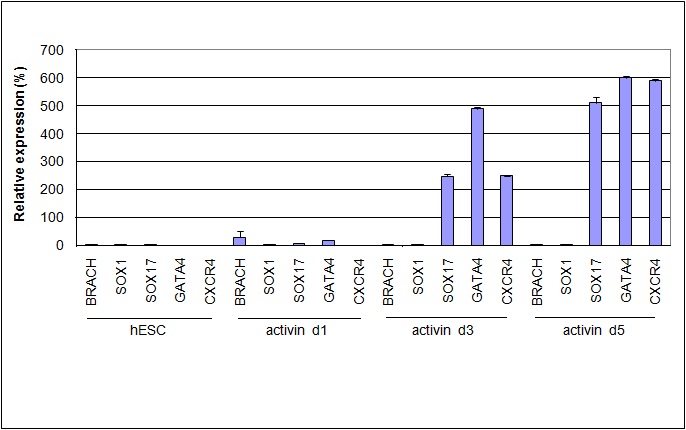

Supplement: Figure S3 — Molecular examination of endodermal differentiation from hESCs over the course of 5 days. RT-qPCR analysis showed that markers of endoderm, including SOX17, GATA4, and CXCR4 become highly expressed at day 3 and day 5 post-differentiation, while BRACHYURY (BRACH), a mesendodermal marker, is expressed transiently at day 1. hESCs have very low expression of endodermal genes. The cells are not expressed SOX1, a neuroectoderm marker throughout the timecourse. X-axis indicates days of endodermal differentiation by activin A; numbers on the Y-axis indicate relative gene expression level, normalized to that of cyclophilinG (CYCG). qPCR was performed using triplicates for each sample, and 3 independent experiments were carried out. Error bars indicate standard derivations which were calculated and reported here using data from one representative experiment. (DOC) [file pone.0017536.s003.doc]

**Figure S4**


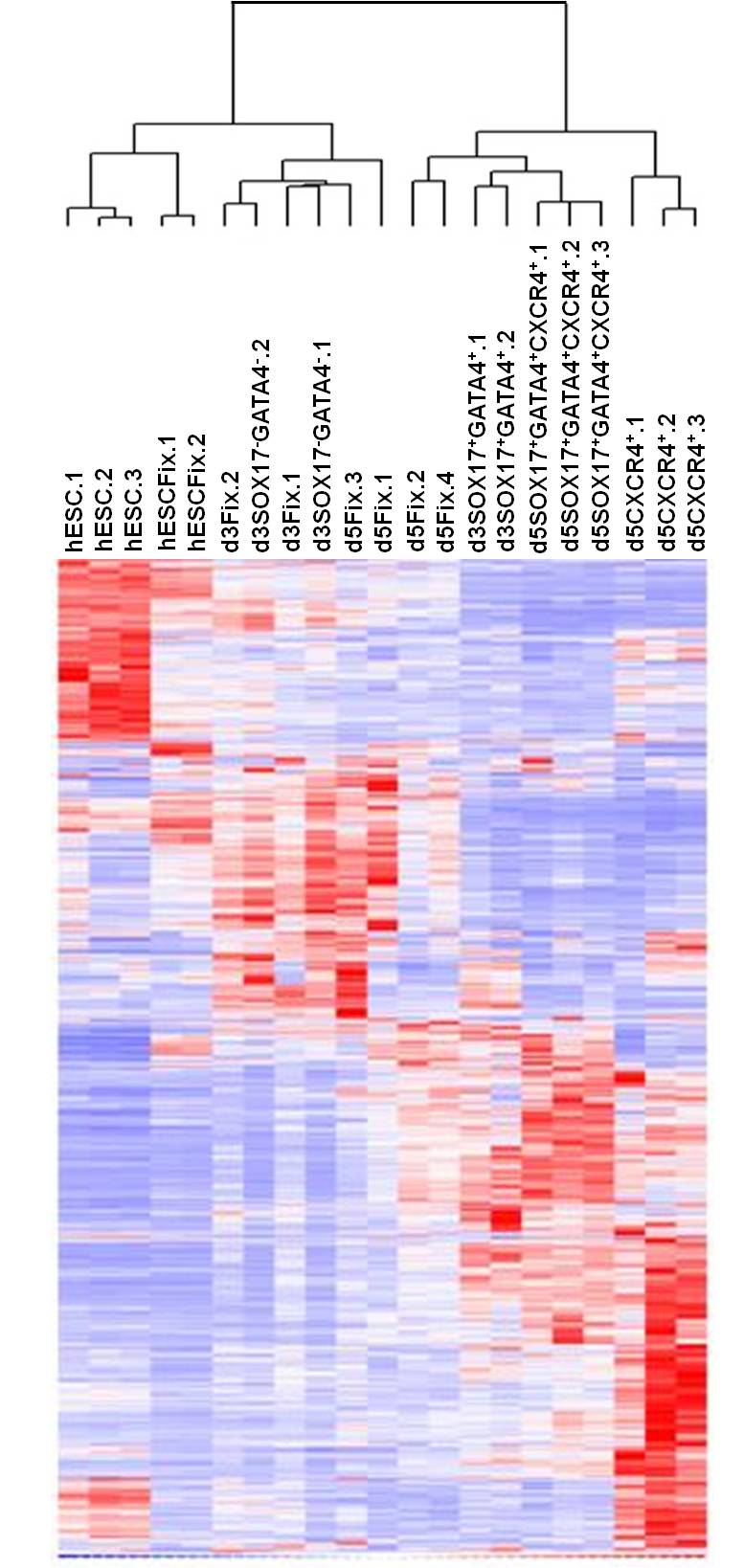

Supplement: Figure S4 — Hierarchical cluster shows that fixatives do not substantially change expression of cell types. We performed hierarchical clustering and found that fixed and unfixed cells cluster together based upon cellular character, and not due to methodology. For example, hESC and d5CXCR4+, which have not been processed, do not cluster together, but clustered with the fixed samples that are biologically similar: hESCs with fixed hESC cells, and d5 CXCR4+ cells with fixed day 5 samples. (DOC) [file pone.0017536.s004.doc]

**Figure S5**


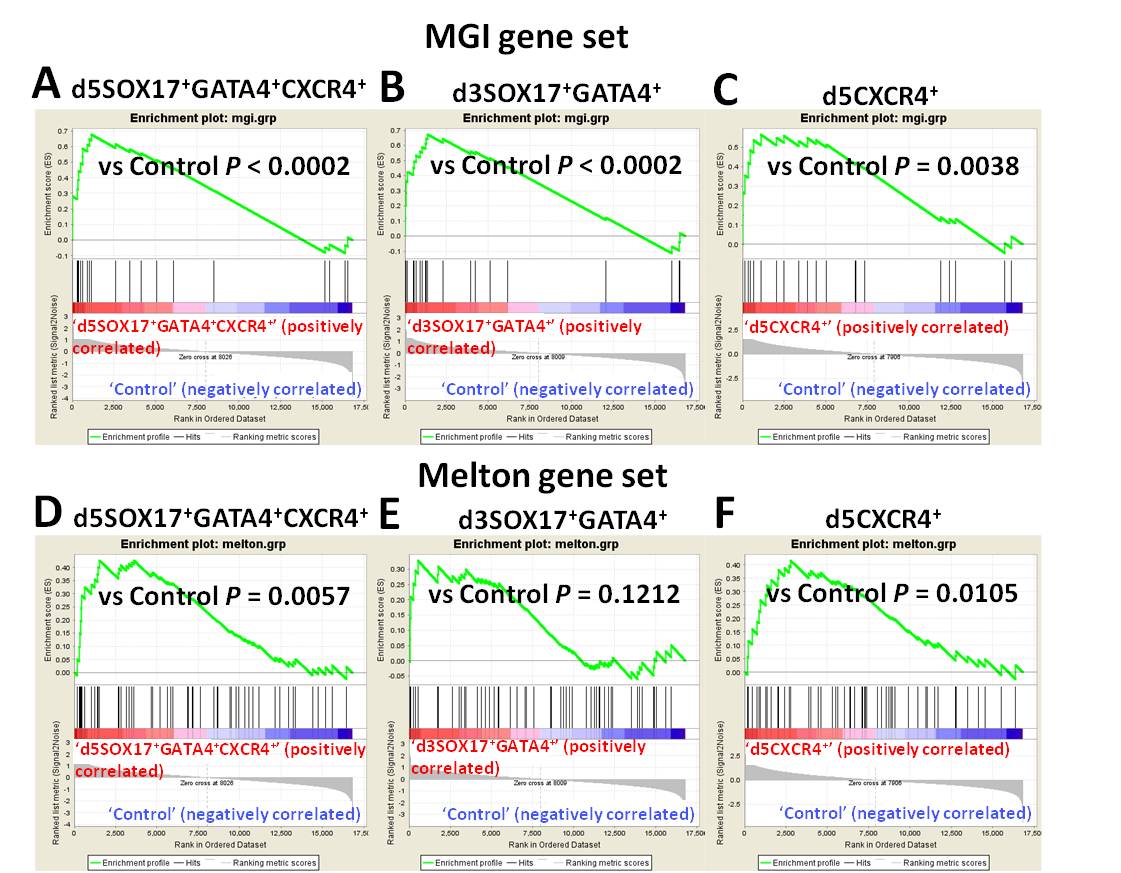

Supplement: Figure S5 — Comparing the definitive endoderm (DE) gene set expression in SOX17+GATA4+CXCR4+ day 5 cells, SOX17+GATA4+ day 3 cells and day 5 CXCR4+ cells using GSEA analysis. We performed GSEA analysis to compare these three populations to the control group, which are all the combined rest samples. While both the MGI DE set and Melton DE set were enriched in both d5 SOX17+GATA4+CXCR4+ and d5 CXCR4+ cells, we observed higher enrichment levels in the d5SOX17+GATA4+CXCR4+ population in both comparisons. MGI: P<0.0002 (A) and P = 0.0038 (C); Melton: P = 0.0057 (D) and P = 0.0105 (F). Interestingly, d3SOX17+GATA4+ cells have similar DE gene sets enrichment to d5 SOX17+GATA4+CXCR4+ cells (B, E). (DOC) [file pone.0017536.s005.doc]
