# Supplementary material for: A New FACS Approach Isolates hESC Derived Endoderm Using Transcription Factors
Source: PLoS One. 2011 Mar 9;6(3):e17536. doi: 10.1371/journal.pone.0017536 (PMC3052315; doi:10.1371/journal.pone.0017536)
Supplement: Table S1 — Primers used for RT-qPCR analysis. (DOC) [file pone.0017536.s006.doc]

**Table S1**. Primers used for RT-qPCR analysis.

| **Gene** | **Primers (Forward / Reverse; 5’-to-3’)** |
| --- | --- |
| *cyclophilin G (CYCG)* | CTTGTCAATGGCCAACAGAGG |
|  | GCCCATCTAAATGAGGAGTTGGT |
| *OCT4* | GAGCAAAACCCGGAGGAGT |
|  | TTCTCTTTCGGGCCTGCAC |
| *BRACHYURY (BRACH)* | TGCTTCCCTGAGACCCAGTT |
|  | GATCACTTCTTTCCTTTGCATCAAG |
| *SOX1* | ATGCACCGCTACGACATGG |
|  | CTCATGTAGCCCTGCGAGTTG |
| *SOX17* | GGCGCAGCAGAATCCAGA |
|  | CCACGACTTGCCCAGCAT |
| *GATA4* | GGAAGCCCAAGAACCTGAAT |
|  | GCTGGAGTTGCTGGAAGC |
| *CXCR4* | CTGTGAGCAGAGGGTCCAG |
|  | ATGAATGTCCACCTCGCTTT |
