# Supplementary material for: A New FACS Approach Isolates hESC Derived Endoderm Using Transcription Factors
Source: PLoS One. 2011 Mar 9;6(3):e17536. doi: 10.1371/journal.pone.0017536 (PMC3052315; doi:10.1371/journal.pone.0017536)
Supplement: Table S2 — Enrichment of top gene categories in the d5 SOX17+GATA4+CXCR4+ cells. (DOC) [file pone.0017536.s007.doc]

**Table S2.** Enrichment of top gene categories in the d5 SOX17^+^GATA4^+^CXCR4^+^ cells.

| **Categories** | **Count** | **Fold Enrichment** | **P Value** |
| --- | --- | --- | --- |
| ***GO Biological Process terms*** |  |  |  |
| GO:0007369~gastrulation | 11 | 8.8 | 4.11E-07 |
| GO:0003007~heart morphogenesis | 10 | 7.7 | 5.54E-06 |
| GO:0007411~axon guidance | 13 | 6.8 | 4.27E-07 |
| GO:0007507~heart development | 20 | 5.2 | 9.63E-09 |
| GO:0035295~tube development | 19 | 4.8 | 7.83E-08 |
| GO:0003002~regionalization | 17 | 4.8 | 4.65E-07 |
| GO:0007389~pattern specification process | 23 | 4.8 | 2.33E-09 |
| GO:0000904~cell morphogenesis involved in differentiation | 21 | 4.8 | 1.41E-08 |
| GO:0048812~neuron projection morphogenesis | 18 | 4.7 | 2.60E-07 |
| GO:0007409~axonogenesis | 16 | 4.6 | 1.87E-06 |
| GO:0048667~cell morphogenesis involved in neuron differentiation | 17 | 4.6 | 1.03E-06 |
| GO:0048858~cell projection morphogenesis | 18 | 4.1 | 1.84E-06 |
| GO:0007420~brain development | 21 | 4.1 | 2.31E-07 |
| ***DE gene sets*** |  |  |  |
| MGI 22 genes | 9 | 22.9 | 1.57E-12 |
| Melton 51 genes | 6 | 6.6 | 3.25E-05 |
