# Supplementary material for: A New FACS Approach Isolates hESC Derived Endoderm Using Transcription Factors
Source: PLoS One. 2011 Mar 9;6(3):e17536. doi: 10.1371/journal.pone.0017536 (PMC3052315; doi:10.1371/journal.pone.0017536)
Supplement: Table S3 — Genes in each enriched category from d5 SOX17+GATA4+CXCR4+ cells. (DOC) [file pone.0017536.s008.doc]

**Table S3**. Genes in each enriched category from d5 SOX17^+^GATA4^+^CXCR4^+^ cells.

| ***GO Biological Process terms*** | **Genes** |
| --- | --- |
| GO:0007369~gastrulation | FGF8,GSC,EYA2,FOXA2,LHX1,NODAL,GATA4,EOMES,SMAD2,NR4A3,MIXL1 |
| GO:0003007~heart morphogenesis | MSX2,DLC1,BMP2,TBX3,TNNC1,NODAL,TBX20,GATA4,COL2A1,TTN |
| GO:0007411~axon guidance | PTPRM,NRP1,OTX2,PAX6,EPHB3,NTN1,SEMA5A,EPHA4,CXCR4,RELN,CNTN4,B3GNT2,UNC5C |
| GO:0007507~heart development | DLC1,BMP2,NRP1,TBX3,TNNC1,NODAL,TBX20,SMAD2,COL2A1,SOX6,TTN,GJA5,MIXL1,MSX2,EDNRA,HHEX,PLCE1,GATA6,GATA4,NRG1 |
| GO:0035295~tube development | DLC1,BMP2,NOG,NRP1,TBX3,FOXA2,NODAL,TBX20,SMAD2,NR4A3,GJA5,FOXP2,EDNRA,SEMA5A,HHEX,CXCR4,GATA6,LHX1,GATA4 |
| GO:0003002~regionalization | HNF1B,NOG,GSC,TBX3,FOXA2,SMAD6,NODAL,TBX20,SIX3,PAX6,SMAD2,HHEX,LHX1,GATA4,ROR2,RELN,SP8 |
| GO:0007389~pattern specification process | HNF1B,NOG,NRP1,GSC,TBX3,FOXA2,NODAL,SMAD6,TBX20,FST,SIX3,PAX6,SMAD2,SEMA5A,EDNRA,HHEX,CXCR4,LHX1,GATA4,ROR2,RELN,SP8,BMP5 |
| GO:0000904~cell morphogenesis involved in differentiation | BMP2,NOG,NRP1,PTPRM,NODAL,OTX2,EOMES,PAX6,EPHB3,NTN1,SLITRK2,NRCAM,SEMA5A,EPHA4,CXCR4,DLX5,RELN,CNTN4,UNC5C,B3GNT2,DSCAM |
| GO:0048812~neuron projection morphogenesis | NRP1,PTPRM,OTX2,LIFR,PAX6,EPHB3,NTN1,SLITRK2,SEMA5A,NRCAM,EPHA4,CXCR4,DLX5,RELN,CNTN4,B3GNT2,UNC5C,DSCAM |
| GO:0007409~axonogenesis | NRP1,PTPRM,OTX2,PAX6,EPHB3,NTN1,SLITRK2,SEMA5A,NRCAM,EPHA4,CXCR4,DLX5,RELN,CNTN4,B3GNT2,UNC5C |
| GO:0048667~cell morphogenesis involved in neuron differentiation | NRP1,PTPRM,OTX2,PAX6,EPHB3,NTN1,SLITRK2,SEMA5A,NRCAM,EPHA4,CXCR4,DLX5,RELN,CNTN4,B3GNT2,UNC5C,DSCAM |
| GO:0048858~cell projection morphogenesis | NRP1,PTPRM,OTX2,LIFR,PAX6,EPHB3,NTN1,SLITRK2,SEMA5A,NRCAM,EPHA4,CXCR4,DLX5,RELN,CNTN4,B3GNT2,UNC5C,DSCAM |
| GO:0007420~brain development | DLC1,HNF1B,NOG,GSC,TBX3,FOXA2,PLXNA2,NODAL,OTX2,SIX3,EOMES,PAX6,FOXP2,HHEX,SLC1A2,DKK1,CXCR4,LHX1,RELN,CNTN4,UNC5C |
| ***DE gene sets*** |  |
| MGI 22 genes | PRDM1, SOX17, DKK1, HHEX, HNF1B, LAMA1, FOXA2, TMPRSS2, EDA |
| Melton 51 genes | SIX3, DLX5, SOX17, GATA3, PAX6, SOX21 |
