# Supplementary material for: A New FACS Approach Isolates hESC Derived Endoderm Using Transcription Factors
Source: PLoS One. 2011 Mar 9;6(3):e17536. doi: 10.1371/journal.pone.0017536 (PMC3052315; doi:10.1371/journal.pone.0017536)
Supplement: Table S4 — Enrichment of top gene categories in the d5 CXCR4+ cells. (DOC) [file pone.0017536.s009.doc]

**Table S4**. Enrichment of top gene categories in the d5 CXCR4^+^ cells.

| **Categories** | **Count** | **Fold Enrichment** | **P Value** |
| --- | --- | --- | --- |
| ***GO Biological Process terms*** |  |  |  |
| GO:0033700~phospholipid efflux | 6 | 24.3 | 2.01E-06 |
| GO:0003007~heart morphogenesis | 14 | 7.8 | 2.26E-08 |
| GO:0009953~dorsal/ventral pattern formation | 11 | 7.4 | 1.84E-06 |
| GO:0007369~gastrulation | 12 | 6.9 | 1.03E-06 |
| GO:0007507~heart development | 23 | 4.3 | 1.72E-08 |
| GO:0003002~regionalization | 19 | 3.9 | 1.86E-06 |
| GO:0007389~pattern specification process | 24 | 3.6 | 1.99E-07 |
| GO:0048646~anatomical structure formation involved in morphogenesis | 29 | 3.3 | 5.03E-08 |
| GO:0009887~organ morphogenesis | 39 | 2.8 | 1.80E-08 |
| GO:0009790~embryonic development | 38 | 2.7 | 6.65E-08 |
| GO:0009653~anatomical structure morphogenesis | 76 | 2.6 | 2.28E-14 |
| GO:0050793~regulation of developmental process | 40 | 2.4 | 6.31E-07 |
| GO:0051239~regulation of multicellular organismal process | 49 | 2.1 | 1.05E-06 |
| GO:0048513~organ development | 90 | 2.1 | 6.18E-12 |
| GO:0007399~nervous system development | 56 | 2.1 | 2.32E-07 |
| GO:0048731~system development | 117 | 2.0 | 5.59E-15 |
| GO:0048856~anatomical structure development | 126 | 2.0 | 3.78E-16 |
| ***DE gene sets*** |  |  |  |
| MGI 22 genes | 7 | 13.3 | 2.38E-08 |
| Melton 51 genes | 8 | 6.6 | 2.93E-06 |
