# Supplementary material for: A New FACS Approach Isolates hESC Derived Endoderm Using Transcription Factors
Source: PLoS One. 2011 Mar 9;6(3):e17536. doi: 10.1371/journal.pone.0017536 (PMC3052315; doi:10.1371/journal.pone.0017536)
Supplement: Table S5 — Genes in each enriched category from d5 CXCR4+ cells. (DOC) [file pone.0017536.s010.doc]

**Table S5**. Genes in each enriched category from d5 CXCR4^+^ cells.

| ***GO Biological Process terms*** | **Genes** |
| --- | --- |
| GO:0033700~phospho-lipid efflux | APOA2,APOA1,APOE,APOC1,ABCA1,ABCG1 |
| GO:0003007~heart morphogenesis | DLC1,BMP2,TNNC1,MYL3,NODAL,TBX20,COL2A1,TTN,MSX2,HAND1,LY6E,SMARCD3,GATA4,FOXC1 |
| GO:0009953~dorsal/ventral pattern formation | NOG,GSC,EVX1,FOXA2,LHX1,SMAD6,TBX20,PAX6,MNX1,HHIP,SP8 |
| GO:0007369~gastrula-tion | FGF8,GSC,EYA2,HAND1,FOXA2,LHX1,NODAL,GATA4,EOMES,AMOT,FOXC1,MIXL1 |
| GO:0007507~heart development | DLC1,BMP2,NRP1,TNNC1,MYL3,NODAL,TBX20,PDLIM3,HSPG2,COL2A1,TTN,MIXL1,MSX2,HHEX,PLCE1,LY6E,HAND1,SMARCD3,GATA6,GATA4,FOXC1,ADAM19,NFATC1 |
| GO:0003002~regionalization | HNF1B,NOG,GSC,EVX1,FOXA2,SMAD6,NODAL,TBX20,PAX6,CYP26A1,HHEX,BTG2,LHX1,GATA4,MNX1,ROR2,RELN,HHIP,SP8 |
| GO:0007389~pattern specification process | HNF1B,NOG,NRP1,GSC,EVX1,FOXA2,SMAD6,NODAL,TBX20,PAX6,CYP26A1,SEMA5A,HHEX,BTG2,CXCR4,LHX1,GATA4,MNX1,ROR2,RELN,HHIP,SP8,BMP5,IHH |
| GO:0048646~anatomi-cal structure formation involved in morphogenesis | DLC1,HNF1B,NOG,NRP1,FOXA2,TBX20,ENPEP,TTN,SEMA5A,SHB,HAND1,CXCR4,LHX1,HMOX1,PLCD1,HS6ST1,SOX17,ANGPT2,IHH,NODAL,EOMES,ARHGAP24,MIXL1,KRT19,EYA2,DLX5,ROR2,FOXC1,TMOD1 |
| GO:0009887~organ morphogenesis | DLC1,NOG,NRP1,CCL2,IRX5,MYL3,TNNC1,TBX20,PAX6,COL2A1,TTN,EPHB2,MSX2,FOXQ1,APOA2,AES,LY6E,HAND1,LHX1,SMARCD3,HLX,GATA4,HHIP,DSCAM,BMP2,GSC,SOCS3,NODAL,EOMES,HSPG2,GAS6,HHEX,LAMA1,EYA2,DLX5,ROR2,FOXC1,GAMT,EDA |
| GO:0009790~embryonic development | DLC1,NOG,HNF1B,FGF8,EVX1,FOXA2,TBX20,CRABP2,PAX6,COL2A1,DLK1,RAI2,MSX2,LY6E,GRIN2B,HAND1,GATA6,LHX1,HLX,FOXF2,GATA4,BMP2,CUBN,GSC,SMAD6,NODAL,EOMES,HSPG2,MIXL1,FOXP2,HHEX,EYA2,DLX5,MNX1,AMOT,ROR2,FOXC1,SP8 |
| GO:0009653~anatomi-cal structure morphogenesis | DLC1,NOG,FGF8,HNF1B,NRP1,FOXA2,TNNC1,EFNA2,CRABP2,TBX20,PAX6,ENPEP,TTN,SHB,APOA2,AES,SMARCD3,APOE,CXCR4,GATA3,HLX,HMOX1,FOXF2,GATA4,HHIP,UNC5C,SOX17,ANGPT2,PITX1,DSCAM,IHH,GSC,SOCS3,NODAL,EOMES,ARHGAP24,MIXL1,SLITRK2,HHEX,KRT19,EYA2,HNF4A,MNX1,ROR2,FOXC1,RELN,EDA,SLC40A1,CCL2,IRX5,MYL3,COL2A1,EPHB3,EPHB2,MSX2,SEMA5A,FOXQ1,LY6E,HAND1,LHX1,HS6ST1,PLCD1,NFATC1,AMHR2,BMP2,HSPG2,FZD5,NTN1,GAS6,LAMA1,EPHA4,DLX5,AMOT,GAMT,SP8,TMOD1 |
| GO:0050793~regulation of developmental process | DLC1,PALM,NOG,LZTS1,NRP1,FOXA2,PAX6,NFKBIA,KIT,ABCA1,EPHB2,GATA6,APOE,LHX1,HMOX1,HLX,GATA4,MBNL3,HIST1H4D,CDC42EP4,ANGPT2,IHH,BMP2,COL4A2,TESC,SOCS3,NODAL,EOMES,CST3,NR0B1,ANKH,NTN1,ABCG1,TRADD,HHEX,LAMA1,DLX5,AMOT,NPPB,IGFBP3 |
| GO:0051239~regulation of multicellular organismal process | GDF3,NOG,NRP1,LZTS1,FOXA2,TNNC1,PAX6,APOA2,APOA1,GRIN2B,GATA6,APOE,HLX,HMOX1,GATA4,ANGPT2,IHH,NODAL,CST3,HHEX,PLCE1,SSTR2,NPPB,MYL4,IRX5,CCL2,MYL3,APOC1,NFKBIA,KIT,EPHB2,ANXA6,LHX1,MBNL3,HIST1H4D,NMU,TESC,COL4A2,BMP2,NTN1,ANKH,TRADD,LAMA1,DIO3,DLX5,ACE2,AMOT,GAMT,IGFBP3 |
| GO:0048513~organ development | DLC1,NOG,HNF1B,NRP1,FOXA2,TNNC1,PLXNA2,EFNA2,CRABP2,TBX20,PDLIM3,PAX6,NFKB2,ENPEP,TTN,SHB,SLC1A2,APOA2,APOA1,AES,GRIN2B,AQP11,APOE,SMARCD3,GATA6,CXCR4,HMOX1,HLX,FOXF2,GATA4,HHIP,UNC5C,SOX17,ANGPT2,PITX1,DSCAM,IHH,GSC,SOCS3,NODAL,RXRG,EOMES,CYP26A1,ARHGAP24,NR0B1,FSHR,MIXL1,HHEX,PLCE1,EYA2,KRT17,KRT16,VAMP5,MNX1,ROR2,FOXC1,RELN,ADAM19,EDA,MYL4,CCL2,BCAT2,IRX5,MYL3,COL2A1,KIT,SRC,EPHB2,MSX2,VCAM1,SEMA5A,FOXQ1,LY6E,HAND1,LHX1,UPK1B,PLCD1,HS6ST1,NFATC1,BMP2,HSPG2,NTN1,GAS6,FOXP2,VWF,LAMA1,DLX5,AMOT,GAMT,BMP5 |
| GO:0007399~nervous system development | DLC1,NOG,HNF1B,PLXNA4,NRP1,LZTS1,EVX1,FOXA2,PLXNA2,FGF17,EFNA2,PAX6,SLC1A2,APOE,CXCR4,HLX,SEMA3E,UNC5C,HHIP,PITX1,AHNAK,DSCAM,GSC,NODAL,ENC1,EOMES,CYP26A1,NR0B1,SLITRK2,HHEX,BTG2,HES4,MNX1,FOXC1,RELN,PLLP,IRX5,KIT,EPHB3,SRC,EPHB2,SEMA5A,LHX1,GAL3ST1,BMP2,TRPC5,HSPG2,NTN4,NTN1,FOXP2,CDKN1C,EPHA4,SEMA6D,ST8SIA4,DLX5,SMPD1 |
| GO:0048731~system development | DLC1,NOG,PLXNA4,TNNC1,PLXNA2,EFNA2,FGF17,CRABP2,PDLIM3,ENPEP,DLK1,TTN,SHB,APOA2,APOA1,GRIN2B,AQP11,GATA6,SMARCD3,APOE,FOXF2,GATA4,UNC5C,PITX1,DSCAM,SOCS3,NODAL,ENC1,RXRG,EOMES,CYP26A1,NR0B1,SLITRK2,HHEX,PLCE1,KRT17,HES4,KRT16,MNX1,VAMP5,ROR2,RELN,STC1,EDA,MYL4,CCL2,MYL3,EPHB3,SRC,EPHB2,SEMA5A,FOXQ1,LHX1,UPK1B,GAL3ST1,BMP2,TRPC5,NTN4,NTN1,ANKH,GAS6,FOXP2,CDKN1C,EPHA4,LAMA1,SEMA6D,DLX5,ST8SIA4,BMP5,HNF1B,LZTS1,NRP1,ARSE,EVX1,FOXA2,TBX20,PAX6,NFKB2,SLC1A2,AES,CXCR4,HMOX1,HLX,SEMA3E,HHIP,SOX17,ANGPT2,AHNAK,IHH,GSC,ARHGAP24,FSHR,MIXL1,EYA2,BTG2,FOXC1,PLLP,ADAM19,BCAT2,IRX5,COL2A1,KIT,MSX2,VCAM1,COL9A2,LY6E,HAND1,PLCD1,HS6ST1,NFATC1,HSPG2,FRZB,VWF,SMPD1,AMOT,GAMT,IGFBP3 |
| GO:0048856~anatomical structure development | DLC1,NOG,PLXNA4,TNNC1,PLXNA2,EFNA2,FGF17,CRABP2,PDLIM3,ENPEP,DLK1,TTN,SHB,APOA2,APOA1,GRIN2B,AQP11,GATA6,SMARCD3,APOE,GATA3,FOXF2,GATA4,UNC5C,PITX1,DSCAM,SOCS3,NODAL,ENC1,RXRG,EOMES,CYP26A1,NR0B1,SLITRK2,HHEX,PLCE1,KRT19,HNF4A,KRT17,KRT16,HES4,MNX1,VAMP5,ROR2,RELN,STC1,EDA,SLC40A1,MYL4,CCL2,MYL3,EPHB3,SRC,EPHB2,SEMA5A,FOXQ1,LHX1,UPK1B,GAL3ST1,BMP2,TRPC5,NTN4,ANKH,NTN1,GAS6,FOXP2,CDKN1C,EPHA4,LAMA1,SEMA6D,DLX5,ST8SIA4,BMP5,TMOD1,FGF8,HNF1B,NRP1,ARSE,LZTS1,EVX1,FOXA2,TBX20,PAX6,NFKB2,SLC1A2,AES,CXCR4,HMOX1,HLX,SEMA3E,HHIP,SOX17,ANGPT2,AHNAK,IHH,GSC,ARHGAP24,FSHR,MIXL1,EYA2,BTG2,FOXC1,PLLP,ADAM19,BCAT2,IRX5,COL2A1,KIT,MSX2,VCAM1,COL9A2,LY6E,HAND1,PLCD1,HS6ST1,NFATC1,AMHR2,HSPG2,FZD5,FRZB,VWF,SMPD1,AMOT,GAMT,SP8,IGFBP3 |
| ***DE gene sets*** |  |
| MGI 22 genes | SOX17, HHEX, HNF1B, LAMA1, FOXA2, TMPRSS2, EDA |
| Melton 51 genes | FOXC1, EVX1, DLX5, SOX17, GATA3, PAX6, IRX5, RIPK4 |
