# Supplementary material for: A New FACS Approach Isolates hESC Derived Endoderm Using Transcription Factors
Source: PLoS One. 2011 Mar 9;6(3):e17536. doi: 10.1371/journal.pone.0017536 (PMC3052315; doi:10.1371/journal.pone.0017536)
Supplement: Table S6 — Enrichment of top gene categories in the d3 SOX17+GATA4+ cells. (DOC) [file pone.0017536.s011.doc]

**Table S6.** Enrichment of top gene categories in the d3 SOX17^+^GATA4^+^ cells.

| **Categories** | **Count** | **Fold Enrichment** | **P Value** |
| --- | --- | --- | --- |
| ***GO Biological Process terms*** |  |  |  |
| GO:0007369~gastrulation | 9 | 11.4 | 1.08E-06 |
| GO:0003002~regionalization | 13 | 5.9 | 2.17E-06 |
| GO:0007389~pattern specification process | 15 | 5.0 | 1.80E-06 |
| GO:0009790~embryonic development | 22 | 3.4 | 1.44E-06 |
| GO:0009653~anatomical structure morphogenesis | 41 | 3.0 | 1.88E-10 |
| GO:0007399~nervous system development | 34 | 2.8 | 1.08E-07 |
| GO:0030154~cell differentiation | 45 | 2.4 | 1.74E-08 |
| GO:0048869~cellular developmental process | 45 | 2.3 | 5.93E-08 |
| GO:0048731~system development | 60 | 2.3 | 1.94E-10 |
| GO:0048513~organ development | 44 | 2.2 | 2.87E-07 |
| GO:0048856~anatomical structure development | 63 | 2.2 | 1.92E-10 |
| GO:0007275~multicellular organismal development | 67 | 2.1 | 5.45E-10 |
| GO:0032502~developmental process | 69 | 1.9 | 4.26E-09 |
| GO:0032501~multicellular organismal process | 80 | 1.7 | 1.56E-07 |
| ***DE gene sets*** |  |  |  |
| MGI 22 genes | 9 | 38 | 8.65E-15 |
| Melton 51 genes | 3 | 5.5 | 0.002 |
