# Supplementary material for: A New FACS Approach Isolates hESC Derived Endoderm Using Transcription Factors
Source: PLoS One. 2011 Mar 9;6(3):e17536. doi: 10.1371/journal.pone.0017536 (PMC3052315; doi:10.1371/journal.pone.0017536)
Supplement: Table S7 — Genes in each enriched category from the d3 SOX17+GATA4+ cells. (DOC) [file pone.0017536.s012.doc]

**Table S7**. Genes in each enriched category from the d3 SOX17^+^GATA4^+^ cells.

| ***GO Biological Process terms*** | **Genes** |
| --- | --- |
| GO:0007369  gastrulation | FGF8,GSC,EYA2,FOXA2,LHX1,GATA4,EOMES,FOXC1,MIXL1 |
| GO:0003002  regionalization | HNF1B,GSC,FOXA2,TBX3,EVX1,SMAD6,CYP26A1,HHEX,SFRP1,LHX1,GATA4,ROR2,HHIP |
| GO:0007389  pattern specification process | HNF1B,GSC,FOXA2,TBX3,EVX1,SMAD6,CYP26A1,SEMA5A,HHEX,SFRP1,CXCR4,LHX1,GATA4,ROR2,HHIP |
| GO:0009790  embryonic development | DLC1,HNF1B,FGF8,BMP2,GSC,EVX1,TBX3,FOXA2,SMAD6,EOMES,COL2A1,MIXL1,HHEX,EYA2,DKK1,SFRP1,LHX1,GATA6,GATA4,ROR2,FOXC1,PRDM1 |
| GO:0009653  anatomical structure morphogenesis | DLC1,HNF1B,FGF8,CCL2,FOXA2,COL2A1,EPHB3,WT1,SEMA5A,SHB,APOA2,CXCR4,LHX1,SMARCD3,GATA4,SOX17,HHIP,B3GNT2,DSCAM,BMP2,GSC,TBX3,COL13A1,OTX2,EOMES,FZD5,ARHGAP24,NTN1,MIXL1,SLITRK2,EPHA4,HHEX,LAMA1,EYA2,DKK1,SFRP1,ROR2,FOXC1,EFNA5,EDA,SLC40A1 |
| GO:0007399  nervous system development | DLC1,SLC5A3,HNF1B,PLXNA4,EVX1,FOXA2,PLXNA2,FGF17,KIT,EPHB3,SEMA5A,PCSK2,SEMA5B,CXCR4,LHX1,SEMA3E,B3GNT2,HHIP,DSCAM,BMP2,GSC,TBX3,OTX2,EOMES,CYP26A1,NTN1,CDKN1C,SLITRK2,HHEX,EPHA4,DKK1,SEMA6D,FOXC1,EFNA5 |
| GO:0030154  cell differentiation | CALCR,HNF1B,EVX1,FOXA2,COL2A1,KIT,EPHB3,WT1,SEMA5A,SEMA5B,SHB,CXCR4,GATA6,LHX1,SMARCD3,UPK1B,SEMA3E,SOX17,HHIP,B3GNT2,DSCAM,SOAT1,BMP2,COL13A1,OTX2,EOMES,CYP26A1,FRZB,FZD5,ARHGAP24,NTN1,MIXL1,CDKN1C,SLITRK2,EPHA4,HHEX,LAMA1,EYA2,SEMA6D,SFRP1,ROR2,FOXC1,EFNA5,PRDM1,EDA |
| GO:0048869  cellular developmental process | CALCR,HNF1B,EVX1,FOXA2,COL2A1,KIT,EPHB3,WT1,SEMA5A,SEMA5B,SHB,CXCR4,GATA6,LHX1,SMARCD3,UPK1B,SEMA3E,SOX17,HHIP,B3GNT2,DSCAM,SOAT1,BMP |
| GO:0048731  system development | DLC1,SLC5A3,HNF1B,ARSE,PLXNA4,EVX1,FOXA2,PLXNA2,FGF17,SHB,APOA2,SMARCD3,GATA6,CXCR4,GATA4,SEMA3E,HHIP,SOX17,DSCAM,GSC,OTX2,EOMES,RXRG,CYP26A1,ARHGAP24,MIXL1,SLITRK2,HHEX,PLCE1,EYA2,ROR2,FOXC1,EFNA5,PRDM1,EDA,CALCR,CCL2,COL2A1,KIT,EPHB3,WT1,PCSK2,SEMA5A,SEMA5B,COL9A2,LHX1,UPK1B,B3GNT2,BMP2,TBX3,COL13A1,FRZB,NTN1,CDKN1C,LAMA1,EPHA4,VWF,DKK1,SEMA6D,SFRP1 |
| GO:0048513  organ development | DLC1,CALCR,HNF1B,CCL2,FOXA2,PLXNA2,COL2A1,KIT,WT1,SEMA5A,SHB,APOA2,CXCR4,GATA6,LHX1,SMARCD3,UPK1B,GATA4,SOX17,HHIP,DSCAM,BMP2,GSC,TBX3,COL13A1,OTX2,EOMES,RXRG,CYP26A1,ARHGAP24,NTN1,MIXL1,HHEX,VWF,LAMA1,PLCE1,EYA2,DKK1,SFRP1,ROR2,FOXC1,EFNA5,PRDM1,EDA |
| GO:0048856  anatomical structure development | DLC1,SLC5A3,FGF8,HNF1B,ARSE,PLXNA4,EVX1,FOXA2,PLXNA2,FGF17,SHB,APOA2,SMARCD3,GATA6,CXCR4,GATA4,SEMA3E,HHIP,SOX17,DSCAM,GSC,OTX2,EOMES, |
| GO:0007275  multicellular organismal development | DLC1,SLC5A3,FGF8,HNF1B,ARSE,PLXNA4,EVX1,FOXA2,PLXNA2,FGF17,SHB,APOA2,SMARCD3,GATA6,CXCR4,GATA4,SEMA3E,HHIP,SOX17,DSCAM,GSC,VANGL1,OTX2,EOMES,RXRG,CYP26A1,ARHGAP24,MIXL1,SLITRK2,FMN2,HHEX,PLCE1,EYA2,ROR2,FOXC1,EFNA5,PRDM1,EDA,CALCR,CCL2,COL2A1,KIT,EPHB3,WT1,PCSK2,SEMA5A,SEMA5B,COL9A2,LHX1,UPK1B,B3GNT2,MBNL3,FZD8,BMP2,TBX3,COL13A1,SMAD6,FZD5,FRZB,NTN1,CDKN1C,LAMA1,EPHA4,VWF,DKK1,SFRP1,SEMA6D |
| GO:0032502  developmental process | DLC1,SLC5A3,FGF8,HNF1B,ARSE,PLXNA4,EVX1,FOXA2,PLXNA2,FGF17,SHB,APOA2,SMARCD3,GATA6,CXCR4,SEMA3E,GATA4,HHIP,SOX17,DSCAM,GSC,VANGL1,OTX2, |
| GO:0032501  multicellular organismal process | DLC1,SLC5A3,FGF8,HNF1B,OR52A1,ARSE,PLXNA4,EVX1,FOXA2,PLXNA2,FGF17,SHB,APOA2,SMARCD3,GATA6,CXCR4,SEMA3E,GATA4,HHIP,SOX17,DSCAM,GSC,VANGL1,MYO3A,CCKBR,TRPA1,OTX2,EOMES,RXRG,CYP26A1,COLEC12,ARHGAP24,MIXL1,SLITRK2,GNAL,HHEX,FMN2,PLCE1,EYA2,NPPB,ROR2,FOXC1,EFNA5,PRDM1,EDA,CALCR,CCL2,OPRK1,KEL,COL2A1,KIT,BDKRB2,EPHB3,WT1,PCSK2,SEMA5A,SEMA5B,COL9A2,LHX1,UPK1B,B3GNT2,MBNL3,GAD1,FZD8,BMP2,TBX3,COL13A1,SMAD6,FZD5,FRZB,NTN1,CDKN1C,VWF,LAMA1,EPHA4,DKK1,SFRP1,SEMA6D,ITGA5,MERTK |
| ***DE gene sets*** |  |
| MGI 22 genes | PRDM1, SOX17, DKK1, HHEX, TMEM46, HNF1B, LAMA1, FOXA2, EDA |
| Melton 51 genes | FOXC1, EVX1, SOX17 |
