# Supplementary material for: A New FACS Approach Isolates hESC Derived Endoderm Using Transcription Factors
Source: PLoS One. 2011 Mar 9;6(3):e17536. doi: 10.1371/journal.pone.0017536 (PMC3052315; doi:10.1371/journal.pone.0017536)
Supplement: Table S8 — Enrichment of top gene categories in the overlapping 197 genes from the d5 SOX17+GATA4+CXCR4+ and d5 CXCR4+ cells. (DOC) [file pone.0017536.s013.doc]

**Table S8.** Enrichment of top gene categories in the overlapping 197 genes from the d5 SOX17^+^GATA4^+^CXCR4^+^ and d5 CXCR4^+^ cells.

| **Categories** | **Count** | **Fold Enrichment** | **P Value** |
| --- | --- | --- | --- |
| ***GO Biological Process terms*** |  |  |  |
| GO:0003002~regionalization | 13 | 6.6 | 6.46E-07 |
| GO:0007389~pattern specification process | 16 | 6.0 | 6.98E-08 |
| GO:0035295~tube development | 13 | 5.9 | 2.07E-06 |
| GO:0000904~cell morphogenesis involved in differentiation | 14 | 5.7 | 9.95E-07 |
| GO:0007420~brain development | 15 | 5.2 | 1.17E-06 |
| GO:0048646~anatomical structure formation involved in morphogenesis | 16 | 4.5 | 2.36E-06 |
| GO:0032989~cellular component morphogenesis | 17 | 4.3 | 2.21E-06 |
| GO:0009790~embryonic development | 22 | 3.9 | 2.10E-07 |
| GO:0009887~organ morphogenesis | 21 | 3.7 | 8.12E-07 |
| GO:0009653~anatomical structure morphogenesis | 40 | 3.3 | 1.73E-11 |
| GO:0042127~regulation of cell proliferation | 24 | 3.0 | 3.16E-06 |
| GO:0007399~nervous system development | 29 | 6.6 | 2.80E-06 |
