# Supplementary material for: A New FACS Approach Isolates hESC Derived Endoderm Using Transcription Factors
Source: PLoS One. 2011 Mar 9;6(3):e17536. doi: 10.1371/journal.pone.0017536 (PMC3052315; doi:10.1371/journal.pone.0017536)
Supplement: Table S9 — Genes in each enriched category with overlapping 197 genes from the d5 SOX17+GATA4+CXCR4+ and d5 CXCR4+ cells. (DOC) [file pone.0017536.s014.doc]

**Table S9**. Genes in each enriched category with overlapping 197 genes from the d5 SOX17^+^GATA4^+^CXCR4^+^ and d5 CXCR4^+^ cells.

| ***GO Biological Process terms*** | **Genes** |
| --- | --- |
| GO:0003002~regionalization | NOG,HNF1B,GSC,FOXA2,NODAL,SMAD6,TBX20,PAX6,HHEX,LHX1,GATA4,RELN,SP8 |
| GO:0007389~pattern specification process | HNF1B,NOG,GSC,FOXA2,SMAD6,NODAL,TBX20,PAX6,  SEMA5A,HHEX,CXCR4,LHX1,GATA4,RELN,SP8,BMP5 |
| GO:0035295~tube development | DLC1,BMP2,NOG,FOXA2,NODAL,TBX20,FOXP2,SEMA5A,HHEX,GATA6,LHX1,CXCR4,GATA4 |
| GO:0000904~cell morphogenesis involved in differentiation | BMP2,NOG,NODAL,EOMES,PAX6,EPHB3,NTN1,SEMA5A,EPHA4,CXCR4,DLX5,RELN,UNC5C,DSCAM |
| GO:0007420~brain development | DLC1,HNF1B,NOG,GSC,FOXA2,NODAL,EOMES,PAX6,FOXP2,HHEX,SLC1A2,CXCR4,LHX1,RELN,UNC5C |
| GO:0048646~anatomical structure formation involved in morphogenesis | DLC1,HNF1B,NOG,FOXA2,NODAL,TBX20,EOMES,ARHGAP24,TTN,MIXL1,SEMA5A,CXCR4,LHX1,DLX5,SOX17,TMOD1 |
| GO:0032989~cellular component morphogenesis | BMP2,NOG,NODAL,EOMES,PAX6,TTN,EPHB3,NTN1,SEMA5A,LAMA1,EPHA4,CXCR4,DLX5,RELN,UNC5C,DSCAM,TMOD1 |
| GO:0009790~embryonic development | DLC1,HNF1B,NOG,FGF8,BMP2,GSC,FOXA2,SMAD6,NODAL,TBX20,EOMES,PAX6,COL2A1,MIXL1,FOXP2,MSX2,HHEX,LHX1,GATA6,DLX5,GATA4,SP8 |
| GO:0009887~organ morphogenesis | DLC1,BMP2,NOG,CCL2,GSC,TNNC1,NODAL,TBX20,EOMES,PAX6,COL2A1,TTN,MSX2,LAMA1,HHEX,APOA2,FOXQ1,LHX1,DLX5,GATA4,DSCAM |
| GO:0009653~anatomical structure morphogenesis | DLC1,NOG,HNF1B,FGF8,CCL2,FOXA2,TNNC1,TBX20,PAX6,COL2A1,EPHB3,TTN,SEMA5A,MSX2,FOXQ1,APOA2,CXCR4,LHX1,GATA3,GATA4,SOX17,UNC5C,DSCAM,BMP2,GSC,NODAL,EOMES,FZD5,ARHGAP24,NTN1,MIXL1,EPHA4,HHEX,LAMA1,HNF4A,DLX5,RELN,SP8,SLC40A1,TMOD1 |
| GO:0042127~regulation of cell proliferation | DLC1,NOG,BMP2,CCL2,CCKBR,NODAL,FGF17,PAX6,ST8SIA1,NFKBIA,KIT,NTN1,FOXP2,VCAM1,MSX2,LAMA1,HHEX,SSTR2,HNF4A,LHX1,DLX5,GATA4,ACE2,ICOSLG |
| GO:0007399~nervous system development | DLC1,HNF1B,NOG,FOXA2,FGF17,PAX6,KIT,EPHB3,SEMA5A,SLC1A2,CXCR4,LHX1,SEMA3E,UNC5C,DSCAM,BMP2,GSC,NODAL,EOMES,NTN4,NTN1,FOXP2,HHEX,EPHA4,SEMA6D,DLX5,ST8SIA4,SMPD1,RELN |
