# Supplementary material for: A New FACS Approach Isolates hESC Derived Endoderm Using Transcription Factors
Source: PLoS One. 2011 Mar 9;6(3):e17536. doi: 10.1371/journal.pone.0017536 (PMC3052315; doi:10.1371/journal.pone.0017536)
Supplement: Table S10 — Enrichment of top gene categories in the unique 241 genes from the d5 CXCR4+ cells. (DOC) [file pone.0017536.s015.doc]

**Table S10.** Enrichment of top gene categories in the unique 241 genes from the d5 CXCR4^+^ cells.

| **Categories** | **Count** | **Fold Enrichment** | **P Value** |
| --- | --- | --- | --- |
| ***GO Biological Process terms*** |  |  |  |
| GO:0030198~extracellular matrix organization | 8 | 5.775777 | 4.61E-04 |
| GO:0014706~striated muscle tissue development | 8 | 5.047738 | 0.001029 |
| GO:0060537~muscle tissue development | 8 | 4.805447 | 0.001371 |
| GO:0048514~blood vessel morphogenesis | 11 | 3.914389 | 5.14E-04 |
| GO:0001568~blood vessel development | 11 | 3.371168 | 0.001596 |
| GO:0001944~vasculature development | 11 | 3.290582 | 0.001915 |
| GO:0009653~anatomical structure morphogenesis | 31 | 1.94456 | 4.99E-04 |
| GO:0065008~regulation of biological quality | 37 | 1.891184 | 1.95E-04 |
| GO:0048513~organ development | 42 | 1.814485 | 1.48E-04 |
| GO:0048731~system development | 56 | 1.804621 | 6.80E-06 |
| GO:0048856~anatomical structure development | 58 | 1.723362 | 1.74E-05 |
| GO:0007275~multicellular organismal development | 62 | 1.624878 | 4.67E-05 |
| GO:0032502~developmental process | 65 | 1.55036 | 1.16E-04 |
