# Supplementary material for: A New FACS Approach Isolates hESC Derived Endoderm Using Transcription Factors
Source: PLoS One. 2011 Mar 9;6(3):e17536. doi: 10.1371/journal.pone.0017536 (PMC3052315; doi:10.1371/journal.pone.0017536)
Supplement: Table S11 — Genes in each enriched category with the unique 241 genes from d5 CXCR4+ cells. (DOC) [file pone.0017536.s016.doc]

**Table S11**. Genes in each enriched category with the unique 241 genes from d5 CXCR4^+^ cells.

| ***GO Biological Process terms*** | **Genes** |
| --- | --- |
| GO:0030198~extracellular matrix organization | COL4A2,EGFLAM,FOXF2,CST3,HSPG2,FOXC1,NFKB2,COL4A6 |
| GO:0014706~striated muscle tissue development | LY6E,HAND1,MYL3,HLX,VAMP5,HSPG2,FOXC1,PITX1 |
| GO:0060537~muscle tissue development | LY6E,HAND1,MYL3,HLX,VAMP5,HSPG2,FOXC1,PITX1 |
| GO:0048514~blood vessel morphogenesis | SHB,HAND1,APOE,HMOX1,AMOT,PLCD1,FOXC1,HS6ST1,ENPEP,ANGPT2,IHH |
| GO:0001568~blood vessel development | SHB,HAND1,APOE,HMOX1,AMOT,PLCD1,FOXC1,HS6ST1,ENPEP,ANGPT2,IHH |
| GO:0001944~vasculature development | SHB,HAND1,APOE,HMOX1,AMOT,PLCD1,FOXC1,HS6ST1,ENPEP,ANGPT2,IHH |
| GO:0009653~anatomical structure morphogenesis | IRX5,MYL3,EFNA2,CRABP2,ENPEP,EPHB2,SHB,AES,LY6E,HAND1,APOE,SMARCD3,HMOX1,HLX,FOXF2,PLCD1,HS6ST1,HHIP,ANGPT2,PITX1,NFATC1,IHH,AMHR2,SOCS3,HSPG2,GAS6,KRT19,MNX1,AMOT,FOXC1,GAMT |
| GO:0065008~regulation of biological quality | C17ORF37,PALM,IRX5,LZTS1,SLC9A5,HPS6,FOXA3,MYL3,KEL,CRABP2,PRDX5,HP,EPHB2,GPX2,LY6E,GRIN2B,APOE,NDRG4,AQP11,HMOX1,PRSS2,SERTAD3,GAL3ST1,F10,EFEMP2,VIL1,CYP26A1,SLC9A3R1,EPB49,ABCG1,LEFTY1,CYBA,NPC2,NXN,AMOT,PLLP,FOXC1 |
| GO:0048513~organ development | IRX5,BCAT2,MYL3,EFNA2,CRABP2,PDLIM3,NFKB2,ENPEP,SRC,EPHB2,SHB,AES,LY6E,HAND1,GRIN2B,AQP11,APOE,SMARCD3,HMOX1,HLX,UPK1B,FOXF2,PLCD1,HS6ST1,HHIP,ANGPT2,PITX1,IHH,NFATC1,SOCS3,HSPG2,CYP26A1,NR0B1,GAS6,KRT17,KRT16,VAMP5,MNX1,AMOT,FOXC1,GAMT,ADAM19 |
| GO:0048731~system development | ARSE,LZTS1,EVX1,EFNA2,CRABP2,PDLIM3,DLK1,ENPEP,NFKB2,SHB,AES,GRIN2B,SMARCD3,APOE,AQP11,HMOX1,HLX,FOXF2,HHIP,ANGPT2,PITX1,AHNAK,IHH,SOCS3,ENC1,CYP26A1,NR0B1,BTG2,KRT17,KRT16,HES4,VAMP5,MNX1,PLLP,FOXC1,ADAM19,IRX5,BCAT2,MYL3,SRC,EPHB2,LY6E,HAND1,UPK1B,PLCD1,HS6ST1,GAL3ST1,NFATC1,TRPC5,HSPG2,ANKH,GAS6,CDKN1C,AMOT,GAMT,IGFBP3 |
| GO:0048856~anatomical structure development | ARSE,LZTS1,EVX1,EFNA2,CRABP2,PDLIM3,DLK1,ENPEP,NFKB2,SHB,AES,GRIN2B,SMARCD3,APOE,AQP11,HLX,HMOX1,FOXF2,HHIP,ANGPT2,PITX1,AHNAK,IHH,SOCS3,ENC1,CYP26A1,NR0B1,KRT19,BTG2,KRT17,KRT16,HES4,VAMP5,MNX1,FOXC1,PLLP,ADAM19,IRX5,BCAT2,MYL3,SRC,EPHB2,LY6E,HAND1,UPK1B,HS6ST1,PLCD1,GAL3ST1,NFATC1,AMHR2,TRPC5,HSPG2,ANKH,GAS6,CDKN1C,AMOT,GAMT,IGFBP3 |
| GO:0007275~multicellular organismal development | LZTS1,ARSE,EVX1,GPR161,EFNA2,CRABP2,PDLIM3,NFKB2,DLK1,ENPEP,SHB,AES,GRIN2B,SMARCD3,APOE,AQP11,HLX,HMOX1,FOXF2,HHIP,ANGPT2,PITX1,AHNAK,IHH,SOCS3,ENC1,CYP26A1,FOSB,NR0B1,LEFTY1,BTG2,KRT17,KRT16,HES4,VAMP5,MNX1,FOXC1,PLLP,ADAM19,IRX5,BCAT2,MYL3,SRC,EPHB2,LY6E,HAND1,NDRG4,UPK1B,HS6ST1,PLCD1,GAL3ST1,NFATC1,AMHR2,TRPC5,HSPG2,ANKH,GAS6,CDKN1C,NXN,AMOT,GAMT,IGFBP3 |
| GO:0032502~developmental process | LZTS1,ARSE,EVX1,HPS6,GPR161,EFNA2,CRABP2,PDLIM3,NFKB2,DLK1,ENPEP,SHB,AES,GRIN2B,SMARCD3,APOE,AQP11,HLX,HMOX1,FOXF2,HHIP,ANGPT2,PITX1,AHNAK,IHH,SOCS3,ENC1,CYP26A1,FOSB,NR0B1,LEFTY1,KRT19,BTG2,KRT17,KRT16,HES4,VAMP5,MNX1,FOXC1,PLLP,ADAM19,IRX5,BCAT2,MYL3,SRC,EPHB2,LY6E,HAND1,NDRG4,UPK1B,HS6ST1,PLCD1,GAL3ST1,NFATC1,AMHR2,TRPC5,HSPG2,ANKH,S100A13,GAS6,CDKN1C,NXN,AMOT,GAMT,IGFBP3 |
