# Supplementary material for: A New FACS Approach Isolates hESC Derived Endoderm Using Transcription Factors
Source: PLoS One. 2011 Mar 9;6(3):e17536. doi: 10.1371/journal.pone.0017536 (PMC3052315; doi:10.1371/journal.pone.0017536)
Supplement: Table S12 — Enrichment of top gene categories in the unique 129 genes from the d5 SOX17+GATA4+CXCR4+ cells. (DOC) [file pone.0017536.s017.doc]

**Table S12.** Enrichment of top gene categories in the unique 129 genes from the d5 SOX17^+^GATA4^+^CXCR4^+^ cells.

| **Categories** | **Count** | **Fold Enrichment** | **P Value** |
| --- | --- | --- | --- |
| ***GO Biological Process terms*** |  |  |  |
| GO:0016337~cell-cell adhesion | 8 | 4.496257 | 0.0019 |
| GO:0007155~cell adhesion | 14 | 3.102418 | 4.73E-04 |
| GO:0022610~biological adhesion | 14 | 3.097992 | 4.79E-04 |
| GO:0010557~positive regulation of macromolecule biosynthetic process | 12 | 2.846255 | 0.0029 |
| GO:0010604~positive regulation of macromolecule metabolic process | 14 | 2.534063 | 0.0030 |
| GO:0031328~positive regulation of cellular biosynthetic process | 12 | 2.717446 | 0.0041 |
| GO:0009891~positive regulation of biosynthetic process | 12 | 2.678346 | 0.0046 |
| GO:0007275~multicellular organismal development | 30 | 1.624302 | 0.0051 |
