# Supplementary material for: A New FACS Approach Isolates hESC Derived Endoderm Using Transcription Factors
Source: PLoS One. 2011 Mar 9;6(3):e17536. doi: 10.1371/journal.pone.0017536 (PMC3052315; doi:10.1371/journal.pone.0017536)
Supplement: Table S13 — Genes in each enriched category with the unique 129 genes from the d5 SOX17+GATA4+CXCR4+ cells. (DOC) [file pone.0017536.s018.doc]

**Table S13.** Genes in each enriched category with the unique 129 genes from the d5 SOX17^+^GATA4^+^CXCR4^+^ cells.

| ***GO Biological Process terms*** | **Genes** |
| --- | --- |
| GO:0016337~cell-cell adhesion | CDH8,NRCAM,PTPRM,PCDHB8,CLDN1,CNTN4,PCDH7,PCDH17 |
| GO:0007155~cell adhesion | FLRT3,PCDHB8,PTPRM,COL21A1,NPNT,NEDD9,PCDH7,PCDH17,CDH8,NRCAM,ARHGAP6,CLDN1,CNTN4,CHL1 |
| GO:0022610~biological adhesion | FLRT3,PCDHB8,PTPRM,COL21A1,NPNT,NEDD9,PCDH7,PCDH17,CDH8,NRCAM,ARHGAP6,CLDN1,CNTN4,CHL1 |
| GO:0010557~positive regulation of macromolecule biosynthetic process | SOAT1,GLIS3,TBX3,YAF2,TICAM1,IL9,SIX3,SMAD2,MAML3,NR4A3,SOX6,SERTAD2 |
| GO:0010604~positive regulation of macromolecule metabolic process | SOAT1,GLIS3,TBX3,IL9,SIX3,SMAD2,NR4A3,SOX6,EDNRA,YAF2,TICAM1,PRDM1,MAML3,SERTAD2 |
| GO:0031328~positive regulation of cellular biosynthetic process | SOAT1,GLIS3,TBX3,YAF2,TICAM1,IL9,SIX3,SMAD2,MAML3,NR4A3,SOX6,SERTAD2 |
| GO:0009891~positive regulation of biosynthetic process | SOAT1,GLIS3,TBX3,YAF2,TICAM1,IL9,SIX3,SMAD2,MAML3,NR4A3,SOX6,SERTAD2 |
| GO:0007275~multicellular organismal development | SLC5A3,CALCR,NPNT,C5,FST,SOX6,GJA5,EDNRA,NRCAM,TNFRSF11B,B3GNT5,SEMA3D,ANGPT1,B3GNT2,NRG1,PTPRM,TBX3,VANG |
